# Genetic analysis of dystonia-related genes in Parkinson's disease

**DOI:** 10.3389/fnagi.2023.1207114

**Published:** 2023-05-26

**Authors:** Yige Wang, Yuwen Zhao, Hongxu Pan, Qian Zeng, Xiaoxia Zhou, Yaqin Xiang, Zhou Zhou, Qian Xu, Qiying Sun, Jieqiong Tan, Xinxiang Yan, Jinchen Li, Jifeng Guo, Beisha Tang, Qiao Yu, Zhenhua Liu

**Affiliations:** ^1^Department of Neurology, Xiangya Hospital, Central South University, Changsha, Hunan, China; ^2^National Clinical Research Center for Geriatric Disorders, Xiangya Hospital, Central South University, Changsha, Hunan, China; ^3^Department of Geriatrics, Xiangya Hospital, Central South University, Changsha, Hunan, China; ^4^Centre for Medical Genetics and Hunan Key Laboratory of Medical Genetics, School of Life Sciences, Central South University, Changsha, Hunan, China; ^5^Key Laboratory of Hunan Province in Neurodegenerative Disorders, Central South University, Changsha, Hunan, China

**Keywords:** Parkinson's disease, dystonia-related genes, rare variants, whole-exome sequencing, whole-genome sequencing

## Abstract

**Objective:**

Parkinson's disease (PD) and dystonia are two closely related movement disorders with overlaps in clinical phenotype. Variants in several dystonia-related genes were demonstrated to be associated with PD; however, genetic evidence for the involvement of dystonia-related genes in PD has not been fully studied. Here, we comprehensively investigated the association between rare variants in dystonia-related genes and PD in a large Chinese cohort.

**Methods:**

We comprehensively analyzed the rare variants of 47 known dystonia-related genes by mining the whole-exome sequencing (WES) and whole-genome sequencing (WGS) data from 3,959 PD patients and 2,931 healthy controls. We initially identified potentially pathogenic variants of dystonia-related genes in patients with PD based on different inheritance models. Sequence kernel association tests were conducted in the next step to detect the association between the burden of rare variants and the risk for PD.

**Results:**

We found that five patients with PD carried potentially pathogenic biallelic variants in recessive dystonia-related genes including *COL6A3* and *TH*. Additionally, we identified 180 deleterious variants in dominant dystonia-related genes based on computational pathogenicity predictions and four of which were considered as potentially pathogenic variants (p.W591X and p.G820S in *ANO3*, p.R678H in *ADCY5*, and p.R458Q in *SLC2A1*). A gene-based burden analysis revealed the increased burden of variant subgroups of *TH, SQSTM1, THAP1*, and *ADCY5* in sporadic early-onset PD, whereas *COL6A3* was associated with sporadic late-onset PD. However, none of them reached statistical significance after the Bonferroni correction.

**Conclusion:**

Our findings indicated that rare variants in several dystonia-related genes are suggestively associated with PD, and taken together, the role of *COL6A3* and *TH* genes in PD is highlighted.

## 1. Introduction

Parkinson's disease (PD), a progressive movement disorder, is the second most common neurodegenerative disorder after Alzheimer's disease. PD is characterized by motor symptoms, including bradykinesia, resting tremor, rigidity, and postural instability, as well as a wide variety of non-motor features, such as olfactory dysfunction, sleep disorders, and dysautonomia (Bloem et al., 2021). As a complex multifactorial disease, PD is caused by a combination of advancing age, genetic, environmental, and lifestyle factors, most of which have not yet been clearly identified (Lim et al., 2019; Bandres-Ciga et al., 2020; Blauwendraat et al., 2020). As another kind of movement disorder, dystonia is characterized by sustained or intermittent muscle contractions causing abnormal, often repetitive, movements, postures, or both. Dystonia can occur in isolation, in combination with other movement disorders, or coexist with a variety of other neurological or systemic manifestations (Albanese et al., 2013). In some conditions, dystonia is more likely a descriptive term rather than a specific diagnosis. The etiology of many forms of dystonia is recognized to be due to a large number of different causes and is still not fully elucidated, but genetic factors are involved unquestionably (Lohmann and Klein, 2013; Balint et al., 2018).

Parkinson's disease and dystonia have overlaps in clinical phenotypes and are both clinically and genetically heterogeneous conditions. Studies estimated that 30% or more of the patients suffering from PD may experience dystonia as a symptom or as a complication of treatment (Tolosa and Compta, 2006; Wickremaratchi et al., 2011). Dystonia is a common early symptom of young-onset PD and sometimes can precede overt parkinsonism, but it can also appear in the middle of advanced stages of PD (Shetty et al., 2019). Conversely, patients suffering from dopa-responsive dystonia (DRD), a specific type of dystonia characterized by lower limb dystonia in childhood with an excellent response to low doses of levodopa, frequently present with parkinsonism. Some DRD patients can even present prominent parkinsonism, leading to clinical difficulty to differentiate between DRD and young-onset PD (Wijemanne and Jankovic, 2015). Especially, dystonia is a prominent symptom for PD patients caused by several genes including *PRKN, PINK1, DJ-1*, and *PLA2G6* (Kasten et al., 2018; Niemann and Jankovic, 2019). Analogously, parkinsonism is commonly seen in subtypes of dystonia caused by genes such as *GCH1, TH, TAF1, ATP1A3*, and *PRKRA* (Phukan et al., 2011; Diez-Fairen et al., 2021).

These observed phenomena indicate a potential correlation between PD and dystonia and suggest the coexistence might be due to overlaps in the genetic background. Therefore, a possible role for dystonia-related genes (abbreviated as the “DYT genes” in this article) in PD is needed to be explored. Our previous research, consistent with other studies from different cohorts, had proven the role of *GCH1*, the most common cause of DRD, in the pathogenesis of PD (Mencacci et al., 2014; Guella et al., 2015; Chen et al., 2016; Pan et al., 2020). However, genetic evidence for the involvement of other DYT genes in PD has not been fully studied. To clarify the correlation between DYT genes and PD, we designed to comprehensively analyze the rare variants of the DYT genes in a large Chinese cohort of patients with PD and healthy controls by mining the whole-exome sequencing (WES) and whole-genome sequencing (WGS) data.

## 2. Materials and methods

### 2.1. Subjects

The enrolled subjects were recruited from Xiangya Hospital, Central South University and other sites of Parkinson's Disease & Movement Disorders Multicenter Database and Collaborative Network in China (PD-MDCNC, http://www.pd-mdcnc.com/). All subjects had undergone basic demographic data collection and peripheral blood sampling to prepare genomic DNA. PD patients are diagnosed according to the Movement Disorder Society (MDS) clinical diagnostic criteria (Postuma et al., 2015), and clinical features have been collected using neuropsychological tests including motor and non-motor manifestations.

In our study, sporadic PD patients were classified as sporadic early-onset PD (sEOPD) or sporadic late-onset PD (sLOPD) depending on the age at onset (AAO) with a cutoff at 50 years old. Controls were confirmed without neurological disorders and obvious family history of neurological disorders. Pathogenic or likely pathogenic variants in the established causative genes of PD were thoroughly screened and excluded from this study according to the previous description (Zhao et al., 2020). Genomic DNA was prepared from peripheral blood leukocytes following standard methods. All subjects or their guardians completed written informed consent, and the study protocol had been approved by the Medical Ethics Committee of Xiangya Hospital, Central South University.

### 2.2. Genotyping and quality control

All subjects were sequenced by WES or WGS depending on our previous or ongoing projects and reanalyzed in the current study. Specifically, familial PD (FPD), sEOPD, and neurological disease-free control group 1 were sequenced by WES, whereas sLOPD and control group 2 were sequenced by WGS. Data generation and quality control procedures for the WES and WGS have been described in detail previously (Pan et al., 2020). Briefly, the sequencing data were first processed following a bioinformatics pipeline (BWA-GATK-ANNOVAR), and subsequently, a series of procedures in quality control was accomplished by the PLINK software (Chang et al., 2015). For individuals with consanguineous family history, homozygosity mapping was performed with PLINK for detecting runs of homozygosity (ROH). Details of genotyping and quality control are shown in Supplementary Methods.

### 2.3. Gene selection

Our analysis included 47 autosomal DYT genes, collected from the Online Mendelian Inheritance in Man (OMIM, https://omim.org/), the Movement Disorder Society Genetic mutation database (MDSGene, https://www.mdsgene.org/), and widely accepted literature (Supplementary Table 1) (Lill et al., 2016; Balint et al., 2018; Wirth et al., 2020; Keller Sarmiento and Mencacci, 2021; Kuipers et al., 2021; Mencacci et al., 2021; Meng et al., 2021; Monfrini et al., 2021; van der Weijden et al., 2021). The protein–protein interactions of the DYT genes and established causative genes of PD assessed by the STRING v11 database (https://string-db.org/) were presented in Supplementary Figure 1 (Szklarczyk et al., 2019). Overall, we selected 19 genes with autosomal recessive (AR) inheritance including *HPCA, TH, PRKRA, COL6A3, MECR, PTS, QDPR, SLC6A3, SLC30A10, CP, DDC, SLC39A14, SLC18A2, SQSTM1, VPS41, COQ8A, VPS11, TSPOAP1*, and *MED27*, 25 genes with autosomal dominant (AD) inheritance including *TOR1A, TUBB4A, THAP1, PNKD, SLC2A1, PRRT2, SGCE, ATP1A3, CIZ1, CACNA1B, ANO3, GNAL, KCTD17, KMT2B, RHOBTB2, KCNA1, CACNA1A, YY1, GNAO1, GNB1, SCN8A, IRF2BPL, NR4A2, EIF2AK2*, and *DRD2*, as well as three genes with both inheritance patterns including *VPS16, SPR*, and *ADCY5*. It should be specially explained that variants of the *GCH1* gene had already been fully analyzed in the current cohorts previously, thus excluded from this study (Pan et al., 2020).

### 2.4. Criteria for rare variants in the DYT genes

**Figure 1:**
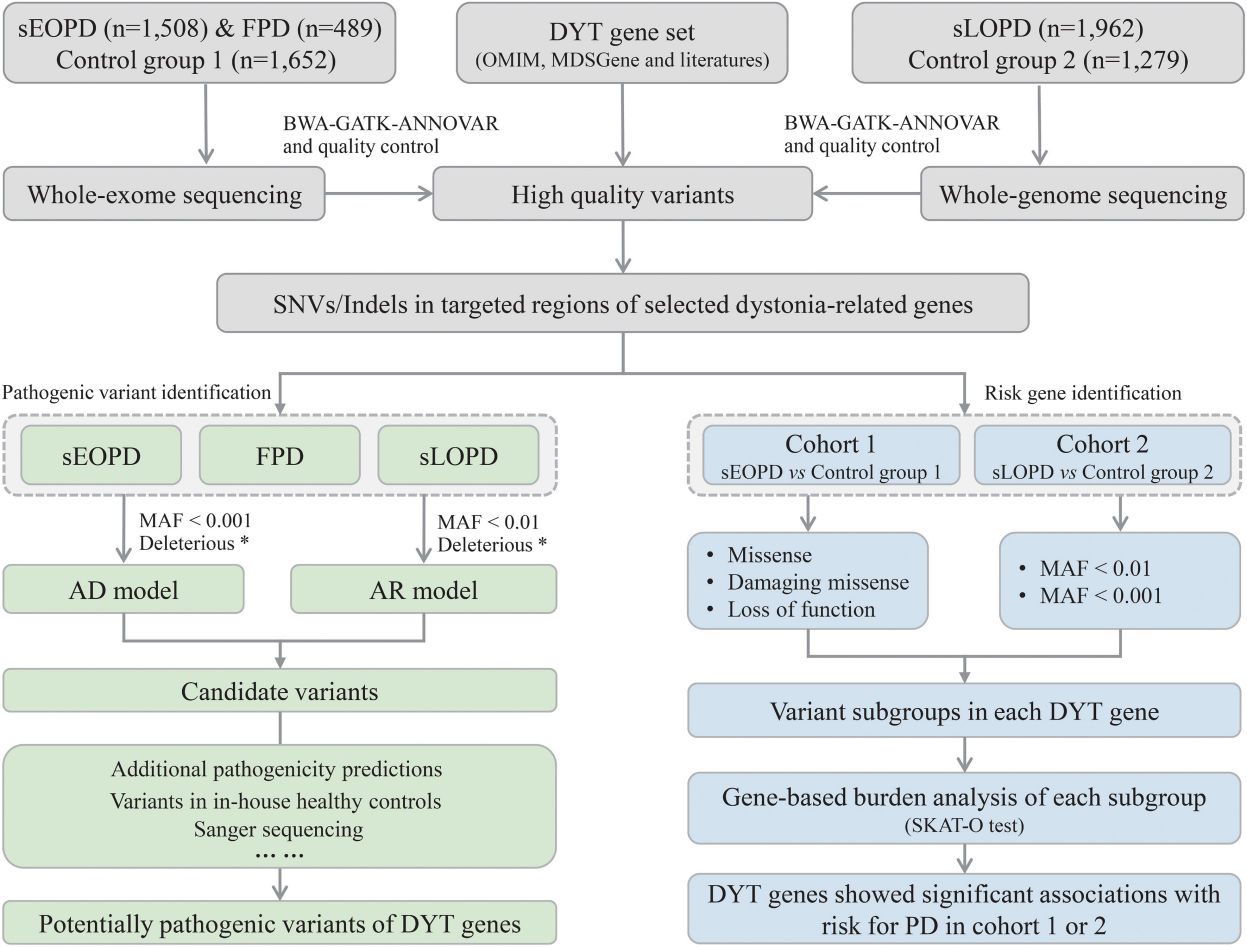
Workflow of the study design. sEOPD, sporadic early-onset Parkinson's disease; FPD, familial Parkinson's disease; sLOPD, sporadic late-onset Parkinson's disease; DYT, dystonia; SNVs, single nucleotide variants; Indels, small insertions/deletions; AD, autosomal dominant; AR, autosomal recessive; MAF, minor allele frequency. ^*^Deleterious: the combination of damaging missense and loss-of-function variants.

The workflow of this study is shown in Figure 1. All high-quality single nucleotide variants and small insertions/deletions (SNVs and Indels) in protein-coding regions of the DYT genes were extracted from processed sequencing data, and then we analyzed from aspects of pathogenic variant identification and risk gene exploration with different subject groups. Minor allele frequency (MAF) of variants was defined by the East Asian population in Genome Aggregation Database (gnomAD) at thresholds of 0.01 and 0.001 (MAF < 0.001 was considered a stricter threshold). ReVe score obtained from VarCards was used to predict pathogenicity for missense variants (Li et al., 2018). Damaging missense (missense variants with ReVe ≥ 0.7) and loss-of-function (stop gain/loss, frameshift, and splicing mutations falling within two base pairs of exon-intron junctions) variants, which have putative devastating effects on proteins, were defined as “deleterious.”

### 2.5. Criteria for potentially pathogenic rare variants in the DYT genes

First, we filtered the rare deleterious variants with two different inheritance patterns under different criteria. For the AR inheritance pattern, filtered variants were deleterious homozygous or putative compound heterozygous states (the compound heterozygous state was not validated to be located on the different DNA strand) with MAF < 0.01 in gnomAD. For AD inheritance, filtered variants were deleterious heterozygous states with MAF < 0.001 in gnomAD. In particular, for genes that reported both recessive and dominant inheritance patterns in dystonia, we, respectively, filtered for different criteria in two conditions.

Subsequently, we scrutinized in-house WES and WGS data of healthy controls to exclude the variants carried by healthy individuals. We gave priority to the filtered variants predicted to be damaging by additional computational pathogenicity predictions including CADD, SIFT, LRT, MutationAssessor, PolyPhen2-HVAR, PolyPhen2-HDIV, and MutationTaster. In addition, we conservatively considered the pathogenicity of the loss-of-function variants, giving thought to variant-specific issues and disease mechanisms in variant interpretation. Variants that survived these criteria were further confirmed by Sanger sequencing and family segregation analysis when the samples were available, and then validated variants were considered potentially pathogenic. We re-evaluated the variants according to standards and guidelines from the American College of Medical Genetics and Genomics (ACMG), thus variants were further classified as pathogenic, likely pathogenic, or uncertain significance (Richards et al., 2015).

### 2.6. Burden analysis

Burden analysis was conducted to evaluate the aggregate association of rare variants with the disease, by optimized sequence kernel association test (SKAT-O) implanted in R (Wu et al., 2011). The variants were categorized into subgroups including missense, damaging missense, loss-of-function, as well as deleterious, with two MAF thresholds, including 0.01 and 0.001.

In the study, we performed SKAT-O for sEOPD and sLOPD compared with the corresponding control group. The SKAT-O test was conducted for each gene independently, considering each variant subgroup separately, with age, sex, and the first five principal components for population stratification as covariates. A *p*-value < 0.001 (0.05/47) was considered statistically significant based on the Bonferroni correction and variant groups not surviving the Bonferroni correction, while uncorrected *p*-values < 0.05 were considered “suggestive.”

## 3. Results

### 3.1. Demographic characteristics

A total of 3,959 PD patients and 2,931 neurological disease-free controls were finally included in the analysis. Cohort 1 consisted of 1,508 sEOPD and 1,652 neurological disease-free controls (Control group 1) sequenced by WES, and cohort 2 consisted of 1,962 sLOPD and 1,279 neurological disease-free controls (Control group 2) sequenced by WGS. In addition, 153 probands of FPD with AR inheritance (ARPD) and 336 probands of FPD with AD inheritance (ADPD) were also sequenced by WES and included in the study. The detailed characteristics of each group are shown in Supplementary Table 2.

### 3.2. PD patients harboring potentially pathogenic variants of recessive DYT genes

For AR inheritance, we detected two deleterious homozygous variants and three pairs of deleterious putative compound heterozygous variants with MAF < 0.01, all of which were predicted to be damaging by multiple computational pathogenicity predictions and not present as biallelic forms in in-house healthy controls (Table 1). The results showed that the *COL6A3* gene was the most frequently mutated DYT gene in PD, and the variants of the *TH* gene were also found in one patient. All the potentially pathogenic variants of recessive DYT genes were confirmed by Sanger sequencing (Supplementary Figure 2), and the detailed phenotypes of carriers are shown in Supplementary Table 3.

**Table 1:** Patients with Parkinson's disease harboring potentially pathogenic variants of recessive dystonia-related genes.

**Sample ID**	**Gene**	**Position (hg19)**	**Ref**	**Alt**	**Exonic function**	**Hom/Het**	**Nucleotide change**	**Amino acid alteration**	**MAF**	**CADD**	**ReVe**	**ACMG**
AR-146	*COL6A3*	chr2:238275918	C	T	Missense	Hom	c.4912G>A	p.A1638T	0.0063/0.0099	29.6	0.851:D	US
EOPD-0488	*COL6A3*	chr2:238283448	G	A	Missense	Het	c.3286C>T	p.R1096C	0.0021/0.0012	23.3	0.728:D	US
		chr2:238266491	G	C	Missense	Het	c.6506C>G	p.P2169R	0.0003/0.0006	27.0	0.766:D	US
EOPD-1304	*COL6A3*	chr2:238283448	G	A	Missense	Het	c.3286C>T	p.R1096C	0.0021/0.0012	23.3	0.728:D	US
		chr2:238249328	G	A	Missense	Het	c.8231C>T	p.T2744M	0/0	27.9	0.889:D	US
EOPD-0766	*COL6A3*	chr2:238277596	G	A	Missense	Het	c.4510C>T	p.R1504W	0/0	24.4	0.856:D	US
		chr2:238270387	G	T	Missense	Het	c.6151C>A	p.P2051T	0/0	21.8	0.763:D	US
LOPD-0390	*TH*	chr11:2192961	G	C	Missense	Hom	c.56C>G	p.S19C	0.0001/NA	26.0	0.777:D	US

MAF, Minor allele frequency in the East Asian population of gnomAD exome database and genome database. NA means the variant was not found in the gnomAD exome or genome database. Hom, Homozygous; Het, Heterozygous; US, uncertain significance.

**Figure 2:**
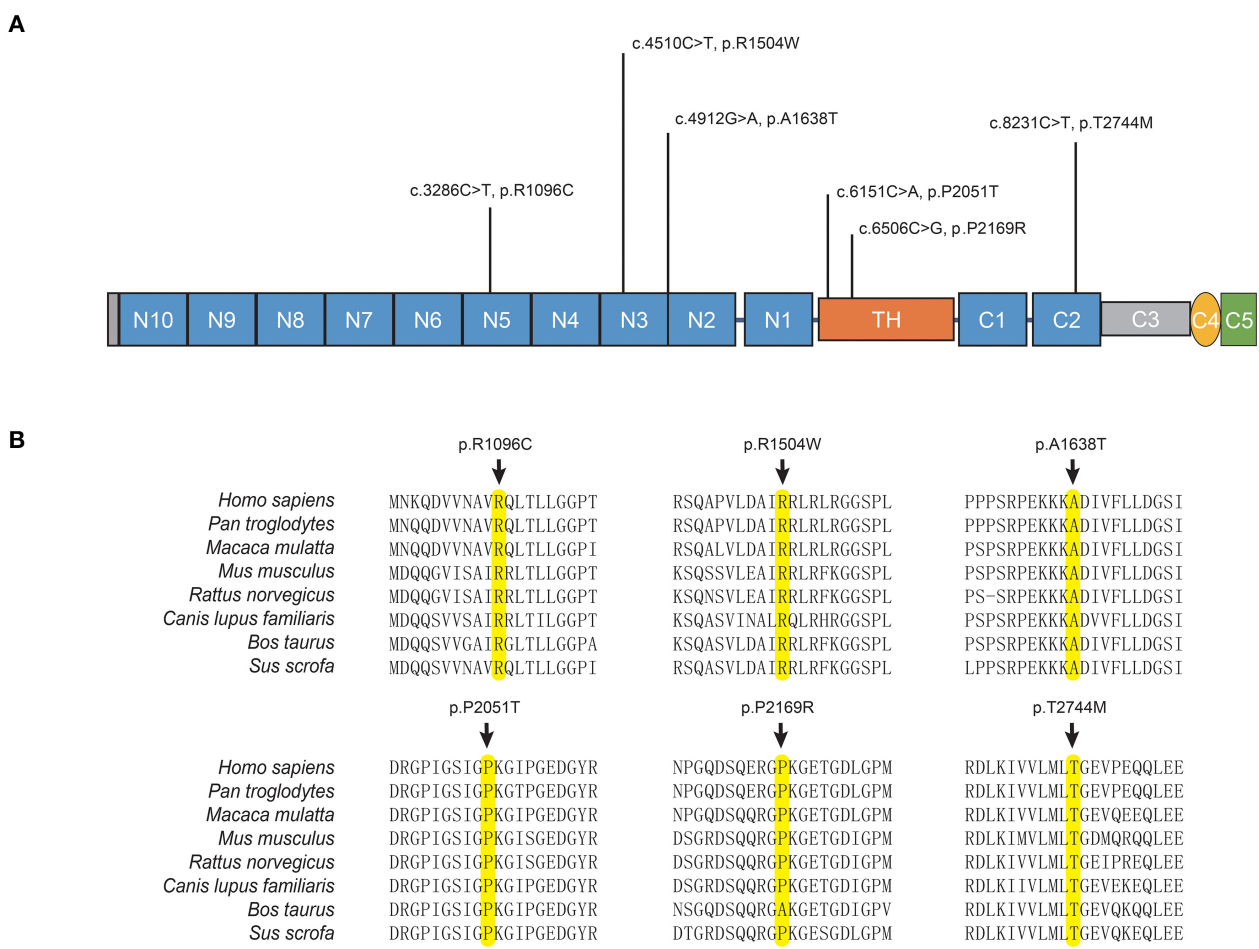
*COL6A3* variants in Parkinson's disease. **(A)** Schematic representation of the domain organization of collagen VI α3 (NP_004360.2) and the localization of identified variants. Collagen VI α3 is composed of a collagen-like triple helical domain (TH) and N and C-terminal globular domains (N1–10 and C1–C5). **(B)** Alignment of multiple collagen VI α3 orthologs. The affected amino acid residues are highlighted in yellow.

The potentially pathogenic variants in *COL6A3* identified in the study are shown in Figure 2. Specifically, we identified a homozygous variant (p.A1638T) in exon 11 of *COL6A3* in the proband of a consanguineous family (AR-146) (Supplementary Figure 3A), located in a 5.36 Mb run of homozygosity (chr2: 237614085-242978914), detected based on homozygosity mapping using the WES data. The patient had an uneventful birth and normal development. At the age of 43, he developed mild rest tremors and clumsiness in his left hand. Subsequently, he had slowed movements in the right hand. A neurological examination performed at the age of 47 showed bilateral motor symptoms including rest tremor, bradykinesia, and rigidity, as well as non-motor symptoms including hyposmia, sleep disturbance, and urinary urgency. It should be noted that the patient presented no sign of dystonia. Brain magnetic resonance imaging (MRI) was reported to be normal, and the results of the electromyogram did not reveal abnormal findings (Supplementary Figure 3B). He responded well to the levodopa-benserazide therapy (500 mg per day) and showed no levodopa-induced dyskinesia. His parents have passed away, and a DNA sample of the unaffected sister was unavailable. In addition, three pairs of heterozygous variants of the *COL6A3* gene were detected in our patients (Table 1), which might form compound heterozygous states and play a role in PD. The patients harboring these variants manifested the typical symptoms of PD with no muscular dystrophy phenotypes or dystonia and were diagnosed as clinically established or probable PD, but one of them developed levodopa-induced dyskinesia.

For the *TH* gene, a novel homozygous variant (p.S19C) was identified in a sporadic patient with an AAO of 59 years. The initial symptoms of the patient were bradykinesia and muscle rigidity of the right lower limb, and over time, postural instability and gait difficulty were gradually manifested. The non-motor symptoms were also remarkable including rapid eye movement sleep behavior disorder (RBD), constipation, cognitive decline, and hyposmia. He was 67 years when he joined the recruitment, and a physical examination showed bilateral rigidity and bradykinesia without any sign of dystonia. Brain MRI was unremarkable. He responded well to dopaminergic therapies (the dose of levodopa-benserazide is 750 mg per day) and showed no levodopa-induced dyskinesia.

### 3.3. PD patients harboring potentially pathogenic variants of dominant DYT genes

For AD inheritance, we initially detected 180 heterozygous variants based on MAF < 0.001 and simultaneously predicted to have putative devastating effects on proteins (missense variants with ReVe ≥ 0.7 or loss-of-function variants) (Supplementary Table 4). After further filtering, four variants were considered potentially pathogenic variants (Table 2). All the variants were not present in gnomAD databases or in-house healthy controls and were loss-of-function variants or predicted to be damaging by multiple computational pathogenicity predictions. Five patients harboring the four potentially pathogenic variants of dominant DYT genes were confirmed by Sanger sequencing (Supplementary Figure 4), and the detailed phenotypes are shown in Supplementary Table 5.

**Table 2:** Patients with Parkinson's disease harboring potentially pathogenic variants of dominant dystonia-related genes.

**Gene**	**Position (hg19)**	**Ref**	**Alt**	**Exonic function**	**Nucleotide change**	**Amino acid alteration**	**MAF**	**CADD**	**ReVe**	**ACMG**	**Sample ID**	**Hom/Het**
*ANO3*	chr11:26621198	G	A	Stopgain	c.1773G>A	p.W591X	NA/NA	41	0.740:D	P	EOPD-0001	Het
*ANO3*	chr11:26669285	G	A	Missense	c.2458G>A	p.G820S	0/0	23.7	0.768:D	LP	EOPD-0190	Het
											EOPD-1469	Het
*ADCY5*	chr3:123044224	C	T	Missense	c.2033G>A	p.R678H	0/0	31	0.854:D	LP	LOPD-1445	Het
*SLC2A1*	chr1:43392818	C	T	Missense	c.1373G>A	p.R458Q	0/NA	26.3	0.812:D	LP	LOPD-0694	Het

MAF, Minor allele frequency in the East Asian population of gnomAD exome database and genome database. NA means the variant was not found in the gnomAD exome or genome database. Hom, Homozygous; Het, Heterozygous; P, Pathogenic; LP, Likely pathogenic.

Two novel variants of *ANO3* (p.W591X and p.G820S) were identified as heterozygous states in our cohort and were carried by three sEOPD patients. One patient harboring the loss-of-function variant p.W591X manifested as a tremor-dominant subtype and developed motor complications including drug-induced dyskinesia and wearing-off, as well as hyposmia and mild depression. The other two patients harboring the p.G820S showed typical motor symptoms whereas the non-motor symptoms were unremarkable. They all responded well to levodopa-benserazide therapy. Cranial MRIs were negative and other laboratory tests were unremarkable. Additionally, we detected a novel heterozygous variant of *ADCY5* (p.R678H) and a reported variant of *SLC2A1* (p.R458Q) in sLOPD patients. The carriers were also diagnosed as PD, with typical motor and non-motor symptoms.

Patients harboring the potentially pathogenic variants were diagnosed as clinically established or probable PD according to the MDS clinical diagnostic criteria (Postuma et al., 2015) and confirmed with no pathogenic or likely pathogenic SNVs/Indels and copy number variations (CNVs) in the established causative genes of PD by WES or WGS and multiplex ligation-dependent probe amplification.

### 3.4. Gene-based burden analysis

In cohort 1, a total of 2,534 variants with a MAF below 0.01 and 2,213 variants with a MAF below 0.001 in the protein-coding regions of 47 DYT genes in cases and controls passed the quality control and were included in the analysis. Similarly, a total of 2,073 variants with a MAF below 0.01 and 1,767 variants with a MAF below 0.001 were included in the analysis of cohort 2.

In cohort 1, we observed suggestive significant associations between loss-of-function variants of *TH* (p = 0.0315) and damaging missense variants of *SQSTM1* (*p* = 0.0212) and the increased risk for sEOPD. Without regard to pathogenicity, missense variants of *SQSTM1* (*p* = 0.0022), *THAP1* (*p* = 0.0322), and *ADCY5* (*p* = 0.0486) also reached a suggestive significance level. In cohort 2, we only detected suggestive associations between damaging missense variants of *COL6A3* (*p* = 0.0346) and the increased risk for sLOPD (Table 3). However, none of these variant subgroups of each DYT gene reached the statistical significance threshold after the Bonferroni correction (*p* > 0.001). The complete results of the burden analysis for each DYT gene are shown in Supplementary Table 6.

**Table 3:** Suggestive associations between the burden of rare variants in dystonia-related genes and the increased risk for Parkinson's disease.

**Gene**	**Cohort**	**Patient subgroup**	**Variant subgroup**	**MAF**<**0.001**				**MAF**<**0.01**			
				**Variants included**	**Case** ^a^	**Control** ^b^	***p*** **-value (SKAT-O)**	**Variants included**	**Case** ^a^	**Control** ^b^	***p*** **-value (SKAT-O)**
*TH*	Cohort 1	sEOPD	LoF	5	8	1	0.0315	5	8	1	0.0315
*SQSTM1*	Cohort 1	sEOPD	Dmis	12	11	3	0.0212	13	14	8	0.1939
*SQSTM1*	Cohort 1	sEOPD	Missense	29	28	12	0.0022	33	37	21	0.0054
*THAP1*	Cohort 1	sEOPD	Missense	5	5	0	0.0322	5	5	0	0.0322
*ADCY5*	Cohort 1	sEOPD	Missense	20	27	18	0.5357	23	45	27	0.0486
*COL6A3*	Cohort 2	sLOPD	Dmis	53	62	25	0.1212	63	143	64	0.0346

^a^Total number of accumulated allele counts of included variants detected in cases of the cohort.

^b^Total number of accumulated allele counts of included variants detected in controls of the cohort.

MAF, Minor allele frequency in the East Asian population of gnomAD exome database and genome database; sEOPD, Sporadic early-onset Parkinson's disease; sLOPD, Sporadic late-onset Parkinson's disease; LoF, Loss-of-function variants; Dmis, Damaging missense variants.

## 4. Discussion

Within two decades, the role of genetics in PD has been emphasized and better understood, but many gaps remain to be filled. We observed similarities in many aspects of PD and dystonia, which sparked our curiosity about the genetic overlaps in these two movement disorders. Consequently, we have comprehensively analyzed the rare variants in coding regions of DYT genes in a large Chinese cohort of PD patients and healthy controls by employing unbiased whole-exome and whole-genome approaches.

To our knowledge, this is the first and largest-ever study to explore the association between rare variants of DYT genes and PD. Firstly, we identified five patients with PD who carried potentially pathogenic biallelic variants in recessive dystonia-related genes, including *COL6A3* and *TH*. Secondly, we detected a total of 180 deleterious variants in dominant dystonia-related genes, four of which were considered potentially pathogenic variants, including p.W591X and p.G820S in *ANO3*, p.R678H in *ADCY5*, and p.R458Q in *SLC2A1*. Thirdly, the burden analysis revealed an increased burden of variant subgroups in *TH, SQSTM1, THAP1*, and *ADCY5* in sEOPD, as well as *COL6A3* in sLOPD, although statistical significance was not achieved after Bonferroni correction. Overall, our study provides evidence that several DYT genes contribute to the pathogenesis of PD, supported by a large sample size and robust methodologies.

The *COL6A3* gene encodes the alpha-3 chain of type VI collagen, which is an extracellular matrix (ECM) protein, playing a pivotal role in the central nervous system (CNS) and participating in various aspects of neurodevelopment and neurodegeneration by regulating autophagy and synaptic plasticity (Dityatev et al., 2010; Neill et al., 2021). Although the physiological role of Col6a3 in the CNS remains poorly understood, emerging data suggest its neuroprotective potential (Cheng et al., 2011; Cescon et al., 2016). Col6a3 was widely expressed throughout the adult mouse brain with the highest mRNA levels in the brainstem and midbrain (Zech et al., 2015). Biallelic loss-of-function mutations of *COL6A3* can cause early-onset segmental isolated dystonia (DYT27) by affecting the EMC in the CNS (Zech et al., 2015). The genetic study of *COL6A3* in PD or other neurodegenerative diseases was insufficient. A previous study conducted in China reported a PD patient without dystonia symptoms carrying compound heterozygous variants in *COL6A3* gene. The study also observed an elevated aggregate variant burden of the *COL6A3* gene in 173 PD patients compared to 200 controls, thereby proposing a potential role of *COL6A3* in PD. However, it is important to note that this evidence is still preliminary (Jin et al., 2021). Here, we reported four patients with PD harboring potentially pathogenic biallelic variants in *COL6A3*, and all the patients showed typical manifestations of PD without dystonia or muscular dystrophy. None of the variants were located in previously reported hotspots for mutation in dystonia (exons 41 and 42), indicating variants in specific regions of *COL6A3* may have an association with PD (Domingo et al., 2016; Jin et al., 2021). We also observed an association between rare damaging variants of the *COL6A3* gene and an increased risk for sLOPD, which replicated the conclusion of the previous study with a larger sample size. As a result, the role of *COL6A3* in PD has been highlighted, although further evidence and mechanism explorations are needed.

*TH* is a well-established causative gene for DRD similar to *GCH1*, and mutations of *TH* and *GCH1*, resulting in deficiencies of the enzymes involved in the dopaminergic synthesis pathway, can present with dystonia and parkinsonism (Lee and Jeon, 2014; Wijemanne and Jankovic, 2015). The association between *GCH1* and PD has been proven previously, which was validated in the Chinese population (Pan et al., 2020). By contrast, the relationship between the *TH* gene and PD is not so clear, due to the limited number of research studies and inconsistencies across the studies (Bademci et al., 2012; Chen et al., 2020; Kawahata and Fukunaga, 2020). Previous studies were mainly focused on common variants of the *TH* gene and utilized a small sample size (Sutherland et al., 2008; Punia et al., 2010). With the rapid advances in genomic technology, subsequent studies performed genetic research between rare variants of *TH* and PD; however, the studies conducted are trivial due to their extremely rare presence with a small sample size (Hertz et al., 2006; Rengmark et al., 2016; Yan et al., 2018). In our cohort, we found a potentially pathogenic homozygous variant of *TH* in a patient with PD without symptoms of DRD, and we also provide suggestive evidence that loss-of-function variants in the *TH* gene might contribute to an increased risk for sEOPD, which help us fully understand the genetic risk for PD conferred by the *TH* gene.

*ANO3* encodes anoctamin 3, which is highly expressed in the brain tissue, specifically in the striatum (Charlesworth et al., 2012). It is a transmembrane protein that belongs to a family of calcium-activated chloride channels and thus may play a role in signal transduction (Pedemonte and Galietta, 2014). Additionally, a previous study has reported an infantile-onset patient manifested dystonia, parkinsonism, and developmental regression, caused by a *de novo* missense variant in *ANO3* (Nelin et al., 2018). However, the exact role of anoctamin 3 remains uncertain, and the two variants found in our patients have not been fully researched in our study and have no other evidence to support the effects yet.

*ADCY5* encodes isoform 5 of adenylyl cyclase, which is highly expressed in medium spiny neurons (MSNs) of the striatum. Adenylyl cyclase is responsible for the conversion of adenosine triphosphate (ATP) to cyclic adenosine-3′,5′-monophosphate (cAMP) and is involved in dopaminergic signaling. Dysfunction of the cAMP pathway can contribute to postsynaptic movement disorders such as dystonia, chorea, and parkinsonism (Abela and Kurian, 2018; Ferrini et al., 2021). A novel variant of *ADCY5* evaluated to be potentially pathogenic was identified in our cohort, and the burden of rare variants in *ADCY5* was increased in sEOPD, which suggested the involvement of *ADCY5* in the pathogenesis of PD, whereas research of the underlying mechanisms is further needed.

Additionally, we identified a variant in *SLC2A1* (p.R458Q) in one PD patient, which was evaluated to be potentially pathogenic. The variant has been recorded as a candidate causal variant of an individual with ataxic gait, global developmental delay, and disrupted sleep, which was included in the ClinVar database. Additionally, a missense variant occurring in the same codon (p.R458W) has been reported in individuals with features of *SLC2A1*-related disorder and has been demonstrated to result in a marked reduction in glucose transport, supporting the functional importance of this position in the protein (Arsov et al., 2012; Tzadok et al., 2014). Therefore, further studies are needed to confirm whether p.R458Q plays a role in PD onset or modification.

The burden analysis also showed the enrichment of extremely rare variants of the *SQSTM1* gene in sEOPD. The *SQSTM1* gene encodes p62, a prototype autophagy receptor, which is commonly found in protein aggregates associated with major neurodegenerative diseases (Deng et al., 2020). It was confirmed to play an essential role in the PINK1/Parkin-mediated mitophagy involved in the pathogenesis of PD (Geisler et al., 2010; Chu, 2019). Mutations in *SQSTM1* contribute to neurodegeneration in amyotrophic lateral sclerosis and frontotemporal dementia (Goode et al., 2016). We have completed an initial exploration and obtained a suggestive but encouraging result about the genetic association between *SQSTM1* and PD, which deserves further investigation. Additionally, *THAP1* was also nominated in the burden analysis, contributing to increased risk for sEOPD. *THAP1* encodes a transcription factor involved in the regulation of gene expression in the nervous system. The reported dysregulated genes by loss of Thap1 are functionally related to neurodevelopment, lysosomal lipid metabolism, myelin, cytoskeleton, synaptic transmission, and gliogenesis (Frederick et al., 2019; Domingo et al., 2021). Our results suggested the involvement of these genes in PD, but much remains to be understood about the underlying mechanisms.

As an exploratory study, limitations are inevitable. First, the DYT genes included in the study had been widely collected by researchers at the preliminary stage of the study, inevitably omitting some latest discovered genes and at the same time, a few controversial genes were incorporated, such as *CACNA1B* and *CIZ1*. The correlation between the two genes and DYT23 was pending confirmation, therefore, the pathogenicity of the variants located in the questioned genes was taken with caution. Besides, we initially detected a large number of variants under AD inheritance, whereas most of them were considered to be uncertain significance due to many aspects such as the reliability and the mechanisms of genes. The conservative interpretation of the variants led to the neglect of certain variants that merit further investigation. Second, we only focused on rare coding SNVs/Indels in this study and did not consider more complex forms of variants, such as CNVs, as well as non-coding variants. The main reason for this decision was the undisclosed role of the DYT genes in PD, which we aimed to explore in the current study. We prioritized SNVs/Indels in coding regions as they were more likely to reveal possible genetic associations. While other types of variants are also important, their complex and potentially indirect mechanisms are currently challenging to elucidate. We will give more attention to the analysis and interpretation of non-coding and complex variants in future studies. Third, the pathogenicity of the discovered genes and variants still requires further validation through family segregation analysis and functional experiments, which have not been comprehensively investigated in our study. Family segregation analysis is a powerful tool for further classification of the identified candidate variants. In our study, we attempted to conduct family segregation analysis using Sanger sequencing in affected or unaffected parents and siblings of the proband whenever feasible. However, we encountered a limitation that most patients had no family history of PD or other neurodegenerative diseases, and unfortunately, DNA samples of their family members were not available. In addition, although it is the largest genetic study of the DYT genes in PD up to now, the sample size was insufficient for rare variants analysis, which may be the cause of little statistical significance detected.

In conclusion, our findings indicated that rare variants in several DYT genes are associated with PD, and the role of *COL6A3* and *TH* genes in PD is highlighted, although further evidence and mechanism explorations are still needed.

## Data availability statement

The data analyzed in this study is subject to the following licenses/restrictions: The data that support the findings of this study are available from the corresponding author upon reasonable request. Requests to access these datasets should be directed to liuzhenhua@csu.edu.cn.

## Ethics statement

The studies involving human participants were reviewed and approved by Medical Ethics Committee of Xiangya Hospital, Central South University. The patients/participants provided their written informed consent to participate in this study.

## Author contributions

YW: conceptualization, data curation, formal analysis, methodology, and writing—original draft. YZ: data curation and writing—review and editing. HP and QZ: data curation and methodology. XZ, YX, and ZZ: data curation. QX, QS, JT, and XY: data curation and funding acquisition. JL, JG, and BT: funding acquisition and writing—review and editing. ZL and QY: project administration, supervision, funding acquisition, and writing—review and editing. All authors contributed to the article and approved the submitted version.
