# Supplementary material for: Genetic analysis of dystonia-related genes in Parkinson's disease
Source: Front Aging Neurosci. 2023 May 26;15:1207114. doi: 10.3389/fnagi.2023.1207114 (PMC10250656; doi:10.3389/fnagi.2023.1207114)
Supplement: Supplementary file 1 [file Data_Sheet_1.docx]

Supplementary Material

Genetic analysis of dystonia-related genes in Parkinson’s disease

Yige Wang, Yuwen Zhao, Hongxu Pan, Qian Zeng, Xiaoxia Zhou, Yaqin Xiang, Zhou Zhou, Qian Xu, Qiying Sun, Jieqiong Tan, Xinxiang Yan, Jinchen Li, Jifeng Guo, Beisha Tang, Qiao Yu*, Zhenhua Liu*

*** Correspondence:** Corresponding Author: liuzhenhua@csu.edu.cn; qiaoyu@csu.edu.cn

# Supplementary Methods

**Genotyping and quality control**

Whole-exome sequencing (WES) was performed on Illumina X10 platform in paired-end 2×150 bp sequencing mode after capturing the whole-exome DNA using SureSelect Human All Exon Kit V6 (Agilent) and preparing sample libraries. The mean WES depth was 129.9X, with a coverage of at least 30X of 94.2% and at least 10X of 99.1%.

Whole-genome sequencing (WGS) was performed on the Illumina NovaSeq 6000 sequencing platform in paired-end 2×150 bp sequencing mode after completing Illumina paired-end DNA library preparation of genomic DNA. The mean WGS depth was 11.9X, with a coverage of at least 8X of 82.1% and at least 4X of 97.0%.

Additionally, we excluded samples with a percentage of bases with a quality score of 30 or higher generated from raw FASTQ file less than 80%.

Subsequently, the raw sequencing data were processed and analyzed with the BWA-GATK-ANNOVAR pipeline, including the sequence alignment by Burrow-Wheeler Aligner (BWA) (Li, 2014), duplicate reads removal by the Picard tool (http://broadinstitute.github.io/picard/), variant calling and filtering by the Genome Analysis Toolkit (GATK) (McKenna et al, 2010), variant annotation by ANNOVAR (Wang et al, 2010; Yang and Wang, 2015). In addition, VarCards (Li et al, 2018a) were also used to annotate the variants and ReVe (Li et al, 2018b) score obtained from VarCards, CADD (Rentzsch et al, 2019), SIFT (Ng and Henikoff, 2003), LRT (Chun and Fay, 2009), MutationAssessor (Reva et al, 2011), PolyPhen2 (Adzhubei et al, 2010) and MutationTaster (Schwarz et al, 2010) were used to predict pathogenicity for missense variants.

To obtain high-quality variants, the PLINK (Chang et al, 2015) software v1.90 was used to accomplish the quality control of individuals and variants. Specifically, individuals showed ambiguous sex (conflicting sex assignment), low genotype call rates (missing rate > 5%), deviating heterozygosity/genotype calls (± 3 standard deviations [SDs]), or cryptic relatedness (identity by descent > 0.15) were excluded. Variants presented low-quality genotypes, low call rates (missing rate > 5%), or departure from Hardy-Weinberg equilibrium (P < 0.0001) were removed. The detailed thresholds of genotype quality we followed were as follows: for WES data, variants with Phred-scaled genotype quality (GQ) score below 20, allele depth (AD) below 5, and reads depth (DP) below 10 were removed; for WGS data, variants with GQ score below 15, AD below 2, and DP below 5 were removed. Furthermore, principal component analysis for population stratification was conducted using independent high-quality variants and main principal component variables for each sample were obtained. Outliers were excluded from further analysis.

**Supplementary References**

Adzhubei, I. A., Schmidt, S., Peshkin, L., Ramensky, V. E., Gerasimova, A., Bork, P., et al (2010) A method and server for predicting damaging missense mutations. *Nat Methods*, 7, 248-9. doi: 10.1038/nmeth0410-248

Chang, C. C., Chow, C. C., Tellier, L. C., Vattikuti, S., Purcell, S. M. and Lee, J. J. (2015) Second-generation PLINK: rising to the challenge of larger and richer datasets. *Gigascience*, 4, 7. doi: 10.1186/s13742-015-0047-8

Chun, S. and Fay, J. C. (2009) Identification of deleterious mutations within three human genomes. *Genome Res*, 19, 1553-61. doi: 10.1101/gr.092619.109

Li, H. (2014) Toward better understanding of artifacts in variant calling from high-coverage samples. *Bioinformatics*, 30, 2843-51. doi: 10.1093/bioinformatics/btu356

Li, J., Shi, L., Zhang, K., Zhang, Y., Hu, S., Zhao, T., et al (2018a) VarCards: an integrated genetic and clinical database for coding variants in the human genome. *Nucleic Acids Res*, 46, D1039-D1048. doi: 10.1093/nar/gkx1039

Li, J., Zhao, T., Zhang, Y., Zhang, K., Shi, L., Chen, Y., et al (2018b) Performance evaluation of pathogenicity-computation methods for missense variants. *Nucleic Acids Res*, 46, 7793-7804. doi: 10.1093/nar/gky678

McKenna, A., Hanna, M., Banks, E., Sivachenko, A., Cibulskis, K., Kernytsky, A., et al (2010) The Genome Analysis Toolkit: a MapReduce framework for analyzing next-generation DNA sequencing data. *Genome Res*, 20, 1297-303. doi: 10.1101/gr.107524.110

Ng, P. C. and Henikoff, S. (2003) SIFT: Predicting amino acid changes that affect protein function. *Nucleic Acids Res*, 31, 3812-4. doi: 10.1093/nar/gkg509

Rentzsch, P., Witten, D., Cooper, G. M., Shendure, J. and Kircher, M. (2019) CADD: predicting the deleteriousness of variants throughout the human genome. *Nucleic Acids Res*, 47, D886-D894. doi: 10.1093/nar/gky1016

Reva, B., Antipin, Y. and Sander, C. (2011) Predicting the functional impact of protein mutations: application to cancer genomics. *Nucleic Acids Res*, 39, e118. doi: 10.1093/nar/gkr407

Schwarz, J. M., Rodelsperger, C., Schuelke, M. and Seelow, D. (2010) MutationTaster evaluates disease-causing potential of sequence alterations. *Nat Methods*, 7, 575-6. doi: 10.1038/nmeth0810-575

Wang, K., Li, M. and Hakonarson, H. (2010) ANNOVAR: functional annotation of genetic variants from high-throughput sequencing data. *Nucleic Acids Res*, 38, e164. doi: 10.1093/nar/gkq603

Yang, H. and Wang, K. (2015) Genomic variant annotation and prioritization with ANNOVAR and wANNOVAR. *Nat Protoc*, 10, 1556-66. doi: 10.1038/nprot.2015.105

# Supplementary Figures and Tables

## Supplementary Figures

Supplementary Figure 1. Protein-protein interaction network of the dystonia-related genes and established causative genes of Parkinson’s disease.

Supplementary Figure 2. Sequencing chromatograms of the potentially pathogenic variants of recessive dystonia-related genes identified in Parkinson’s disease.

Supplementary Figure 3. Pedigree chart and brain MRI of patient with Parkinson’s disease harboring homozygous variant in *COL6A3*. (A) Pedigree chart of the patient with Parkinson’s disease harboring homozygous variant c.4912G>A (p.A1638T) in *COL6A3*. Black filled symbols denote PD patients and the arrow indicates the proband. Circles indicate women and squares indicate men, and diagonal lines indicate deceased individuals. Var: variant. (B) MRI of the patient harboring p.A1638T in *COL6A3*.

Supplementary Figure 4. Sequencing chromatograms of the potentially pathogenic variants of dominant dystonia-related genes identified in Parkinson’s disease.


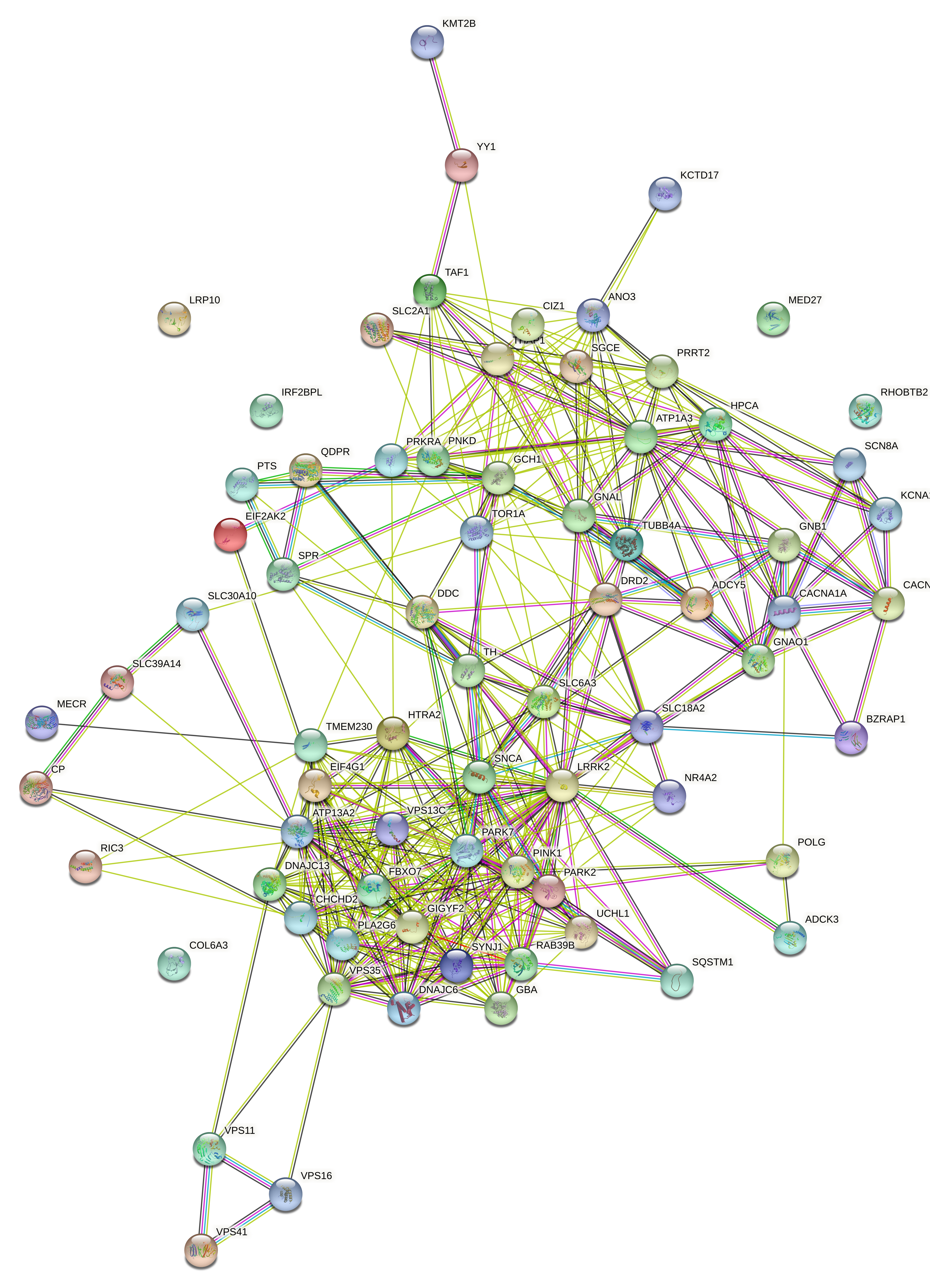


**Supplementary Figure 1.** Protein-protein interaction network of the dystonia-related genes and established causative genes of Parkinson’s disease.


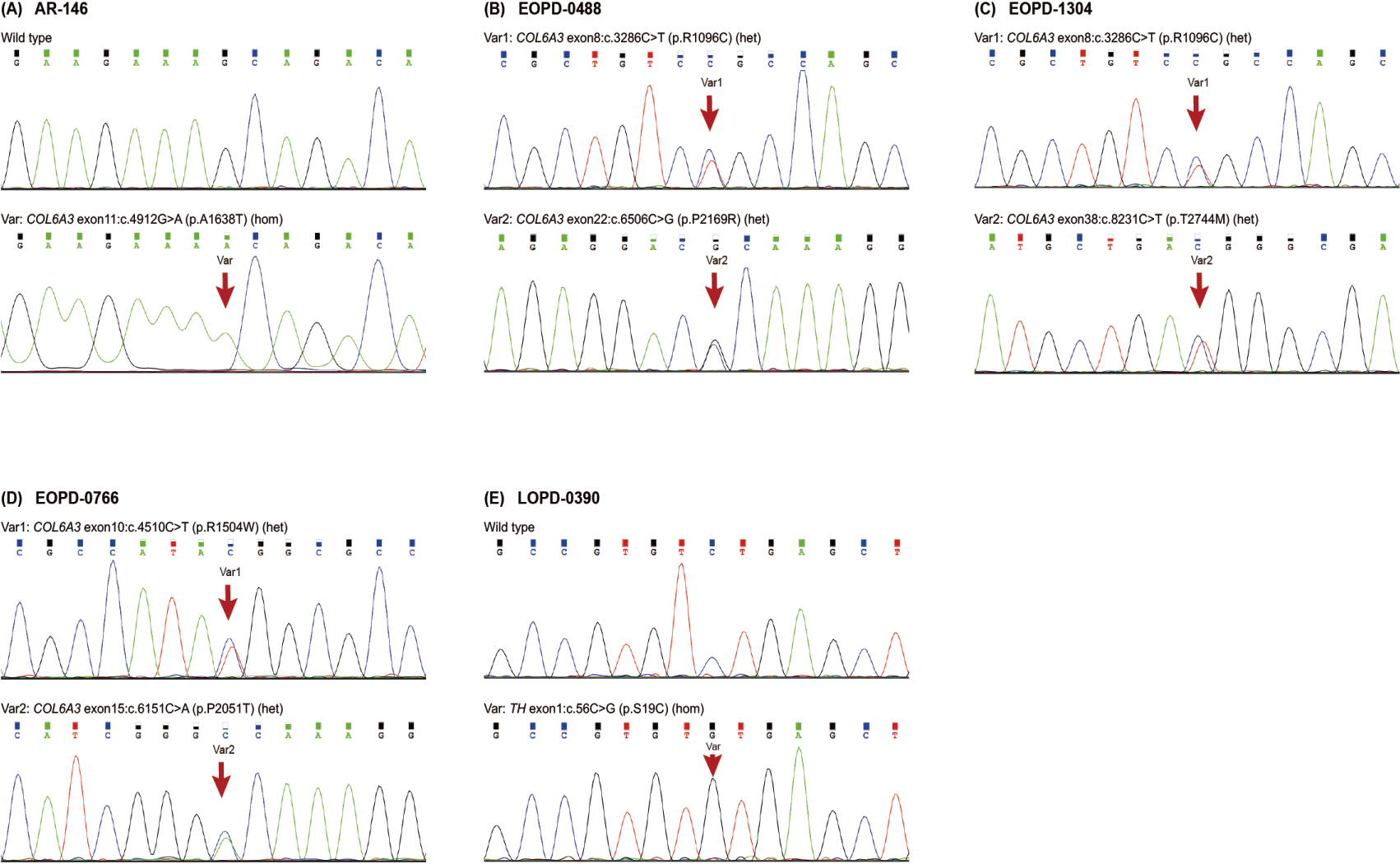


**Supplementary Figure 2.** Sequencing chromatograms of the potentially pathogenic variants of recessive dystonia-related genes identified in Parkinson’s disease.


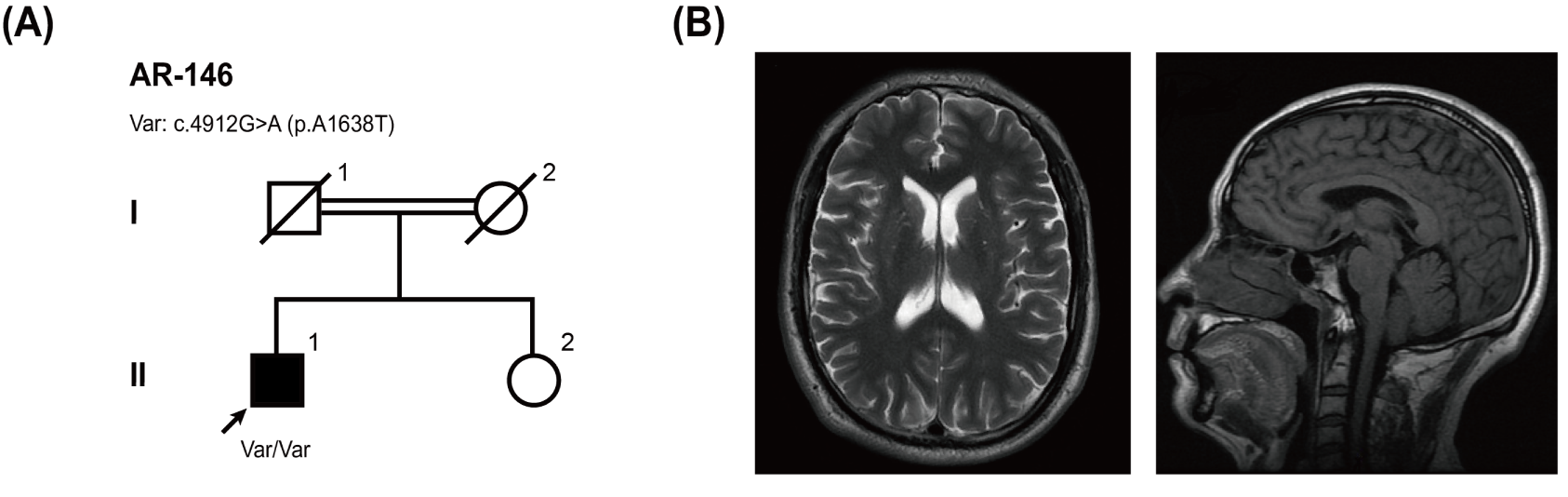


**Supplementary Figure 3.** Pedigree chart and brain MRI of patient with Parkinson’s disease harboring homozygous variant in *COL6A3*. (A) Pedigree chart of the patient with Parkinson’s disease harboring homozygous variant c.4912G>A (p.A1638T) in *COL6A3*. Black filled symbols denote PD patients and the arrow indicates the proband. Circles indicate women and squares indicate men, and diagonal lines indicate deceased individuals. Var: variant. (B) MRI of the patient harboring p.A1638T in *COL6A3*.


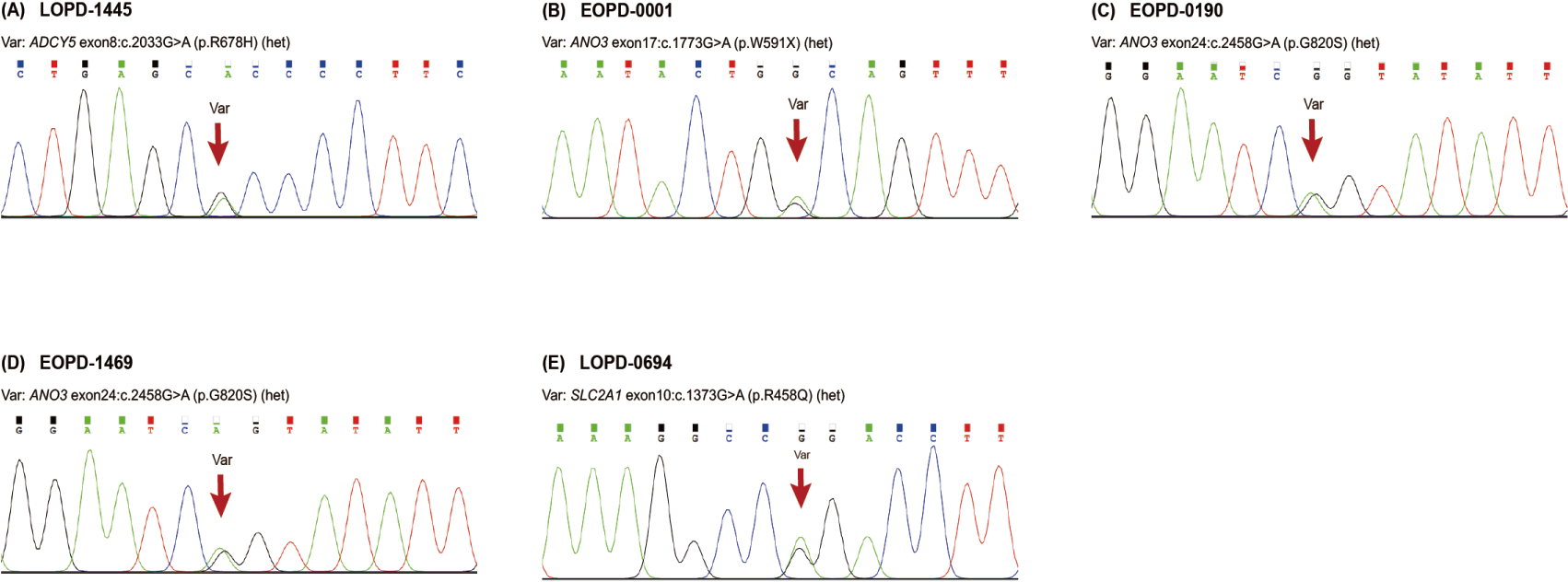


**Supplementary Figure 4.** Sequencing chromatograms of the potentially pathogenic variants of dominant dystonia-related genes identified in Parkinson’s disease.

## Supplementary Tables

Supplementary Table 1. Summary of targeted dystonia-related genes in this study.

Supplementary Table 2. Basic demographic characteristics of included subjects.

Supplementary Table 3. The clinical phenotype of patients with Parkinson’s disease harboring biallelic potentially pathogenic variants of recessive dystonia-related genes.

Supplementary Table 4. Rare deleterious variants of dominant dystonia-related genes identified in Parkinson’s disease.

Supplementary Table 5. The clinical phenotype of patients with Parkinson’s disease harboring monoallelic potentially pathogenic variants of dominant dystonia-related genes.

Supplementary Table 6. Burden analyses of each dystonia-related gene in Parkinson’s disease.

**Supplementary Table 1.** Summary of targeted dystonia-related genes in this study.

| **Gene** | **Protein** | **Inheritance** | **Dystonia phenotype** | **OMIM** | **Cytogenetic location** | **Genomic coordinates (hg19)** | **Refseq** | **Reference** |
| --- | --- | --- | --- | --- | --- | --- | --- | --- |
| *TOR1A* | Torsin-1A | AD | DYT1 | 605204 | 9q34.11 | chr9:132575221-132586441 | NM_000113 | OMIM |
| *HPCA* | Neuron-specific calcium-binding protein hippocalcin | AR | DYT2 | 142622 | 1p35.1 | chr1:33352098-33360247 | NM_002143 | OMIM |
| *TUBB4A* | Tubulin beta-4A chain | AD | DYT4 | 602662 | 19p13.3 | chr19:6494330-6502330 | NM_006087 | OMIM |
| *TH* | Tyrosine hydroxylase | AR | DYT5b | 191290 | 11p15.5 | chr11:2185159-2193035 | NM_199292 | OMIM |
| *THAP1* | THAP domain-containing protein 1 | AD | DYT6 | 609520 | 8p11.21 | chr8:42691817-42698474 | NM_018105 | OMIM |
| *PNKD* | Probable hydrolase PNKD | AD | DYT8 | 609023 | 2q35 | chr2:219135115-219211516 | NM_015488 | OMIM |
| *SLC2A1* | Solute carrier family 2, facilitated glucose transporter member 1 | AD | DYT9/  DYT18 | 138140 | 1p34.2 | chr1:43391046-43424847 | NM_006516 | OMIM |
| *PRRT2* | Proline-rich transmembrane protein 2 | AD | DYT10 | 614386 | 16p11.2 | chr16:29823409-29827202 | NM_001256442 | OMIM |
| *SGCE* | Epsilon-sarcoglycan | AD | DYT11 | 604149 | 7q21.3 | chr7:94214536-94285521 | NM_001099400 | OMIM |
| *ATP1A3* | Na+/K+ transporting ATPase subunit alpha-3 | AD | DYT12 | 182350 | 19q13.2 | chr19:42470734-42498428 | NM_152296 | OMIM |
| *PRKRA* | Interferon-inducible double-stranded RNA-dependent protein kinase activator A | AR | DYT16 | 603424 | 2q31.3 | chr2:179296141-179315958 | NM_003690 | OMIM |
| *CIZ1* | Cip1-interacting zinc finger protein | AD | DYT23 | 611420 | 9q34.11 | chr9:130928344-130954383 | NM_001131016 | OMIM |
| *CACNA1B* | Voltage-dependent N-type calcium channel subunit alpha-1B | AD | DYT23 | 601012 | 9q34.3 | chr9:140772241-141019076 | NM_000718 | OMIM |
| *ANO3* | Anoctamin-3 | AD | DYT24 | 610110 | 11p14.3-p14.2 | chr11:26353678-26684836 | NM_031418 | OMIM |
| *GNAL* | Guanine nucleotide-binding protein G(olf) subunit alpha | AD | DYT25 | 139312 | 18p11.21 | chr18:11689014-11885683 | NM_182978 | OMIM |
| *KCTD17* | BTB/POZ domain-containing protein KCTD17 | AD | DYT26 | 616386 | 22q12.3 | chr22:37447779-37459430 | NM_024681 | OMIM |
| *COL6A3* | Collagen alpha-3(VI) chain | AR | DYT27 | 120250 | 2q37.3 | chr2:238232655-238322850 | NM_004369 | OMIM |
| *KMT2B* | Histone-lysine N-methyltransferase 2B | AD | DYT28 | 606834 | 19q13.12 | chr19:36208921-36229779 | NM_014727 | OMIM |
| *MECR* | Enoyl-[acyl-carrier-protein] reductase, mitochondrial | AR | DYT29 | 608205 | 1p35.3 | chr1:29519385-29557454 | NM_016011 | OMIM |
| *VPS16* | Vacuolar protein sorting-associated protein 16 homolog | AD/AR | DYT30 | 608550 | 20p13 | chr20:2821373-2847378 | NM_022575 | OMIM |
| *SPR* | Sepiapterin reductase | AD/AR | Unassigned | 182125 | 2p13.2 | chr2:73114512-73119289 | NM_003124 | MDSGene |
| *PTS* | 6-pyruvoyl tetrahydrobiopterin synthase | AR | Unassigned | 612719 | 11q23.1 | chr11:112097088-112104695 | NM_000317 | MDSGene |
| *QDPR* | Dihydropteridine reductase | AR | Unassigned | 612676 | 4p15.32 | chr4:17488016-17513857 | NM_000320 | MDSGene |
| *SLC6A3* | Sodium-dependent dopamine transporter | AR | Unassigned | 126455 | 5p15.3 | chr5:1392905-1445543 | NM_001044 | MDSGene |
| *SLC30A10* | Zinc transporter 10 | AR | Unassigned | 611146 | 1q41 | chr1:220087606-220101993 | NM_018713 | MDSGene |
| *CP* | Ceruloplasmin | AR | Unassigned | 117700 | 3q24-q25 | chr3:148890290-148939832 | NM_000096 | MDSGene |
| *ADCY5* | Adenylate cyclase type 5 | AD/AR | Unassigned | 600293 | 3q21.1 | chr3:123001143-123167392 | NM_183357 | PMID: 30237473 |
| *DDC* | Aromatic-L-amino-acid decarboxylase | AR | Unassigned | 107930 | 7p12.2-p12.1 | chr7:50526134-50628768 | NM_000790 | PMID: 30237473 |
| *SLC39A14* | Metal cation symporter ZIP14 | AR | Unassigned | 608736 | 8p21.3 | chr8:22225050-22280249 | NM_001135153 | PMID: 30237473 |
| *SLC18A2* | Synaptic vesicular amine transporter | AR | Unassigned | 193001 | 10q25.3 | chr10:119000584-119038941 | NM_003054 | PMID: 33564903 |
| *SQSTM1* | Sequestosome-1 (p62) | AR | Unassigned | 601530 | 5q35 | chr5:179247842-179265077 | NM_003900 | PMID: 33564903 |
| *RHOBTB2* | Rho-related BTB domain-containing protein 2 | AD | Unassigned | 607352 | 8p21.3 | chr8:22853361-22877710 | NM_001160037 | PMID: 33564903 |
| *KCNA1* | Potassium voltage-gated channel subfamily A member 1 | AD | Unassigned | 176260 | 12p13.32 | chr12:5019073-5027422 | NM_000217 | PMID: 33564903 |
| *CACNA1A* | Voltage-dependent P/Q-type calcium channel subunit alpha-1A | AD | Unassigned | 601011 | 19p13.13 | chr19:13317256-13617274 | NM_001127222 | PMID: 33564903 |
| *VPS41* | Vacuolar protein sorting-associated protein 41 homolog | AR | Unassigned | 605485 | 7p14.1 | chr7:38763543-38948800 | NM_014396 | PMID: 33564903 |
| *YY1* | Transcriptional repressor protein YY1 | AD | Unassigned | 600013 | 14q32.2 | chr14:100705102-100745371 | NM_003403 | PMID: 33564903 |
| *COQ8A* | Atypical kinase COQ8A, mitochondrial | AR | Unassigned | 606980 | 1q42.13 | chr1:227127938-227175246 | NM_020247 | PMID: 33564903 |
| *GNAO1* | Guanine nucleotide-binding protein G(o) subunit alpha | AD | Unassigned | 139311 | 16q13 | chr16:56225251-56382111 | NM_138736 | PMID: 33564903 |
| *GNB1* | Guanine nucleotide-binding protein G(I)/G(S)/G(T) subunit beta-1 | AD | Unassigned | 139380 | 1p36.33 | chr1:1716725-1822526 | NM_002074 | PMID: 33564903 |
| *SCN8A* | Sodium channel protein type 8 subunit alpha | AD | Unassigned | 600702 | 12q13.13 | chr12:51985020-52206648 | NM_014191 | PMID: 33564903 |
| *IRF2BPL* | Probable E3 ubiquitin-protein ligase IRF2BPL | AD | Unassigned | 611720 | 14q24.3 | chr14:77490886-77495042 | NM_024496 | PMID: 33564903 |
| *VPS11* | Vacuolar protein sorting-associated protein 11 homolog | AR | Unassigned | 608549 | 11q23.3 | chr11:118938493-118952688 | NM_021729 | PMID: 33452836 |
| *NR4A2* | Nuclear receptor subfamily 4 group A member 2 | AD | Unassigned | 601828 | 2q24.1 | chr2:157180944-157189287 | NM_006186 | PMID: 31922365 |
| *EIF2AK2* | Interferon-induced, double-stranded RNA-activated protein kinase | AD | Unassigned | 176871 | 2p22.2 | chr2:37332284-37384190 | NM_001135651 | PMID: 33236446 |
| *DRD2* | D (2) dopamine receptor | AD | Unassigned | 126450 | 11q23.2 | chr11:113280317-113346001 | NM_000795 | PMID: 33200438 |
| *TSPOAP1* | Peripheral-type benzodiazepine receptor-associated protein 1 | AR | Unassigned | 610764 | 17q22 | chr17:56378588-56406152 | NM_004758 | PMID: 33539324 |
| *MED27* | Mediator of RNA polymerase II transcription subunit 27 | AR | Unassigned | 605044 | 9q34.13 | chr9:134735497-134955274 | NM_004269 | PMID: 33443317 |

AD: Autosomal dominant; AR: Autosomal recessive; OMIM: Online Mendelian Inheritance in Man; MDSGene: the Movement Disorder Society Genetic mutation database; PMID: PubMed unique identifier.

**Supplementary Table 2.** Basic demographic characteristics of included subjects.

| **Characteristics** | **ADPD probands**  (n = 336) | **ARPD probands**  (n = 153) | **Cohort 1** | |  | **Cohort 2** | |
| --- | --- | --- | --- | --- | --- | --- | --- |
|  |  |  | **sEOPD**  (n = 1,508) | **Control group 1**  (n = 1,652) |  | **sLOPD**  (n = 1,962) | **Control group 2**  (n = 1,279) |
| Age, years | 56.79±10.99 | 61.41±10.55 | 50.16±6.99 | 62.03±12.59 |  | 66.76±7.08 | 62.32±7.11 |
| Age at onset, years | 51.59±10.75 | 55.92±11.31 | 44.19±5.47 | - |  | 61.88±6.93 | - |
| Sex (male/female) | 184/152 | 80/73 | 827/681 | 795/857 |  | 984/978 | 613/666 |
| Genotyping method | WES | WES | WES | WES |  | WGS | WGS |

ADPD: Autosomal dominant Parkinson’s disease; ARPD: Autosomal recessive Parkinson’s disease; sEOPD: Sporadic early-onset Parkinson’s disease; sLOPD: Sporadic late-onset Parkinson’s disease; WES: Whole-exome sequencing; WGS: Whole-genome sequencing.

**Supplementary Table 3.** The clinical phenotype of patients with Parkinson’s disease harboring biallelic potentially pathogenic variants of recessive dystonia-related genes.

| **Case** | **AR-146** | **EOPD-0488** | **EOPD-1304** | **EOPD-0766** | **LOPD-0390** |
| --- | --- | --- | --- | --- | --- |
| DYT gene | *COL6A3* | *COL6A3* | *COL6A3* | *COL6A3* | *TH* |
| Sex | M | F | F | M | F |
| Age at onset(yrs) | 43 | 45 | 43 | 40 | 59 |
| Age at examination(yrs) | 49 | 49 | 47 | 50 | 67 |
| Disease duration(yrs) | 6 | 4 | 4 | 10 | 8 |
| Hoehn-Yahr stage | 2.5 | 2.5 | 2 | 3 | 3 |
| Bradykinesia | + | + | + | + | + |
| Resting tremor | + | – | + | + | – |
| Rigidity | + | + | + | + | + |
| Postural instability | – | + | – | + | + |
| UPDRS-III | 40 | 38 | 29 | 52 | 47 |
| Motor subtype | TD | PIGD | TD | TD | PIGD |
| Hyposmia | + | – | – | + | + |
| Depression | – | – | – | – | – |
| Urinary urgency | + | + | – | – | – |
| Constipation | – | + | + | + | + |
| Cognitive decline | – | – | – | – | + |
| MMSE | 28 | 29 | 29 | 29 | 11 |
| Sleep disturbance | + | + | + | + | – |
| RBD | – | – | + | – | + |
| Freezing gait | – | + | + | + | + |
| Response to levodopa | good | good | good | good | good |
| Wearing off | – | + | + | + | + |
| Dyskinesia | – | – | – | + | – |
| PD diagnosis * | EstPD | ProPD | EstPD | EstPD | EstPD |

* PD diagnosis means cases were classified in accordance with the Movement Disorder Society clinical diagnostic criteria for Parkinson’s disease. –: absent; +: present; yrs: years; PIGD: postural instability and gait disorders; TD: Tremor dominant; PD: Parkinson’s disease; EstPD: Established PD; ProPD: Probable PD.

**Supplementary Table 4.** Rare deleterious variants of dominant dystonia-related genes identified in Parkinson’s disease.

| **Gene** | **Hom/Het** | **Position** | **Ref** | **Alt** | **Nucleotide change** | **Amino acid alteration** | **Exonic function** | **MAF ^a^** | **ReVe** |
| --- | --- | --- | --- | --- | --- | --- | --- | --- | --- |
| *ADCY5* | Het | chr3:123008792 | C | T | c.3337G>A | p.E1113K | Missense | 0.0001/- | 0.933:D |
| *ADCY5* | Het | chr3:123010114 | C | T | c.3173G>A | p.R1058H | Missense | 0/0 | 0.841:D |
| *ADCY5* | Het | chr3:123014996 | C | T | c.2998G>A | p.A1000T | Missense | -/- | 0.848:D |
| *ADCY5* | Het | chr3:123015025 | G | A | c.2969C>T | p.T990M | Missense | 0/- | 0.886:D |
| *ADCY5* | Het | chr3:123019076 | C | T | c.2791G>A | p.G931R | Missense | 0/- | 0.971:D |
| *ADCY5* | Het | chr3:123044288 | C | T | c.1969G>A | p.A657T | Missense | 0.0002/0.0006 | 0.738:D |
| *ADCY5* | Het | chr3:123049786 | G | T | c.1596C>A | p.D532E | Missense | -/- | 0.874:D |
| *ADCY5* | Het | chr3:123051437 | A | C | c.1492T>G | p.F498V | Missense | -/- | 0.998:D |
| *ADCY5* | Het | chr3:123071401 | A | C | c.1162T>G | p.C388G | Missense | -/- | 0.947:D |
| *ADCY5* | Het | chr3:123166269 | A | G | c.1124T>C | p.L375P | Missense | -/- | 0.949:D |
| *ANO3* | Het | chr11:26463518 | C | T | c.100C>T | p.R34W | Missense | -/- | 0.728:D |
| *ANO3* | Het | chr11:26552783 | T | C | c.769T>C | p.W257R | Missense | 0.00005798/- | 0.909:D |
| *ANO3* | Het | chr11:26552867 | C | T | c.853C>T | p.R285C | Missense | 0/- | 0.929:D |
| *ANO3* | Het | chr11:26563516 | C | T | c.1055C>T | p.P352L | Missense | 0.0001/- | 0.835:D |
| *ANO3* | Het | chr11:26563588 | A | G | c.1127A>G | p.Y376C | Missense | 0.0002/0.0006 | 0.925:D |
| *ANO3* | Het | chr11:26569010 | G | T | c.1202G>T | p.W401L | Missense | 0.0001/- | 0.892:D |
| *ANO3* | Het | chr11:26569064 | A | G | c.1256A>G | p.Y419C | Missense | 0/- | 0.925:D |
| *ANO3* | Het | chr11:26620415 | G | A | c.1541G>A | p.R514H | Missense | 0/0 | 0.771:D |
| *ANO3* | Het | chr11:26681908 | G | A | c.2863G>A | p.E955K | Missense | -/- | 0.882:D |
| *ATP1A3* | Het | chr19:42479840 | G | A | c.2204C>T | p.A735V | Missense | -/- | 0.803:D |
| *ATP1A3* | Het | chr19:42482089 | G | C | c.1942C>G | p.R648G | Missense; Splicing | -/- | 0.731:D |
| *ATP1A3* | Het | chr19:42482129 | G | C | c.1902C>G | p.I634M | Missense | -/- | 0.788:D |
| *CACNA1A* | Het | chr19:13319666 | A | C | c.6684T>G | p.Y2228X | Stopgain | -/- | 0.323:T |
| *CACNA1A* | Het | chr19:13319748 | C | T | c.6602G>A | p.R2201Q | Missense | 0.00009872/- | 0.713:D |
| *CACNA1A* | Het | chr19:13320159 | G | A | c.6493C>T | p.R2165C | Missense | 0.0003/0 | 0.776:D |
| *CACNA1A* | Het | chr19:13322946 | A | G | c.6274T>C | p.S2092P | Missense | -/- | 0.798:D |
| *CACNA1A* | Het | chr19:13323254 | A | T | c.6133T>A | p.W2045R | Missense | -/- | 0.858:D |
| *CACNA1A* | Het | chr19:13335577 | G | A | c.5635C>T | p.R1879W | Missense | 0.00006168/- | 0.896:D |
| *CACNA1A* | Het | chr19:13340903 | C | T | c.5521G>A | p.A1841T | Missense | 0/- | 0.911:D |
| *CACNA1A* | Het | chr19:13365938 | C | T | c.4726G>A | p.A1576T | Missense | -/- | 0.829:D |
| *CACNA1A* | Het | chr19:13365962 | C | T | c.4702G>A | p.E1568K | Missense | -/- | 0.920:D |
| *CACNA1A* | Het | chr19:13368183 | T | C | c.4571A>G | p.Y1524C | Missense | -/- | 0.807:D |
| *CACNA1A* | Het | chr19:13386700 | A | G | c.3953T>C | p.I1318T | Missense | -/- | 0.906:D |
| *CACNA1A* | Het | chr19:13441069 | A | G | c.1334T>C | p.I445T | Missense | 0/- | 0.715:D |
| *CACNA1A* | Het | chr19:13441142 | G | A | c.1261C>T | p.R421W | Missense | 0/- | 0.889:D |
| *CACNA1A* | Het | chr19:13443725 | C | T | c.1213G>A | p.A405T | Missense | 0/- | 0.807:D |
| *CACNA1A* | Het | chr19:13445207 | - | TT | c.1182_1183insAA | p.W395Nfs*8 | Frameshift | -/- | - |
| *CACNA1A* | Het | chr19:13565974 | C | A | c.346G>T | p.A116S | Missense | 0.0002/0.0006 | 0.704:D |
| *CACNA1B* | Het | chr9:140777307 | G | A | c.502G>A | p.V168I | Missense | 0/0.0006 | 0.807:D |
| *CACNA1B* | Het | chr9:140807666 | C | T | c.565C>T | p.R189X | Stopgain | -/- | 0.580:T |
| *CACNA1B* | Het | chr9:140850209 | G | A | c.1130G>A | p.R377Q | Missense | 0/- | 0.855:D |
| *CACNA1B* | Het | chr9:140865849 | C | T | c.1348C>T | p.R450C | Missense | 0/- | 0.864:D |
| *CACNA1B* | Het | chr9:140865895 | G | T | c.1394G>T | p.R465L | Missense | 0.00005813/- | 0.768:D |
| *CACNA1B* | Het | chr9:140917581 | C | T | c.2386C>T | p.R796W | Missense | -/- | 0.879:D |
| *CACNA1B* | Het | chr9:140917746 | C | G | c.2551C>G | p.H851D | Missense | -/- | 0.701:D |
| *CACNA1B* | Het | chr9:140941882 | G | C | c.3575G>C | p.G1192A | Missense | -/- | 0.879:D |
| *CACNA1B* | Het | chr9:140948377 | T | C | c.3887T>C | p.F1296S | Missense | -/- | 0.978:D |
| *CACNA1B* | Het | chr9:140952664 | G | T | c.4270G>T | p.D1424Y | Missense | -/- | 0.908:D |
| *CACNA1B* | Het | chr9:140953592 | C | G | c.4535C>G | p.S1512C | Missense | 0.00005801/- | 0.818:D |
| *CACNA1B* | Het | chr9:140953627 | G | A | c.4570G>A | p.A1524T | Missense | 0.00005802/- | 0.966:D |
| *CACNA1B* | Het | chr9:140954142 | G | A | c.4621G>A | p.V1541I | Missense | 0.0002/- | 0.799:D |
| *CACNA1B* | Het | chr9:140990988 | G | A | c.5147G>A | p.R1716Q | Missense | 0.0001/- | 0.901:D |
| *CACNA1B* | Het | chr9:141012490 | G | A | c.5870G>A | p.R1957H | Missense | 0.00006042/0 | 0.818:D |
| *CACNA1B* | Het | chr9:141013121 | G | A | c.5931G>A | p.M1977I | Missense | 0/- | 0.735:D |
| *CACNA1B* | Het | chr9:141014636 | C | G | c.6050C>G | p.P2017R | Missense | -/- | 0.804:D |
| *CACNA1B* | Het | chr9:141014708 | G | A | c.6122G>A | p.R2041H | Missense | 0/- | 0.725:D |
| *CACNA1B* | Het | chr9:141014739 | C | - | c.6153delC | p.H2052Tfs*116 | Frameshift | -/- | - |
| *CACNA1B* | Het | chr9:141015154 | C | T | c.6310C>T | p.R2104C | Missense | 0/- | 0.834:D |
| *CACNA1B* | Het | chr9:141015155 | G | A | c.6311G>A | p.R2104H | Missense | 0/- | 0.796:D |
| *CACNA1B* | Het | chr9:141015233 | G | A | c.6389G>A | p.C2130Y | Missense | -/- | 0.851:D |
| *CACNA1B* | Het | chr9:141015250 | C | T | c.6406C>T | p.R2136C | Missense | 0/- | 0.825:D |
| *CACNA1B* | Het | chr9:141016186 | C | T | c.6755C>T | p.S2252F | Missense | -/- | 0.737:D |
| *CACNA1B* | Het | chr9:141016359 | G | A | c.6928G>A | p.V2310M | Missense | 0.00005804/0 | 0.775:D |
| *CACNA1B* | Het | chr9:141016368 | G | A | c.6937G>A | p.G2313S | Missense | 0/0 | 0.863:D |
| *CACNA1B* | Het | chr9:141016449 | T | C | c.7018T>C | p.X2340Q | Stoploss | -/- | 0.545:T |
| *CIZ1* | Het | chr9:130929102 | T | C | c.2423A>G | p.Y808C | Missense | -/- | 0.898:D |
| *CIZ1* | Het | chr9:130941312 | G | A | c.1174C>T | p.Q392X | Stopgain | -/- | 0.431:T |
| *CIZ1* | Het | chr9:130942803 | C | T | c.683-1G>A | Splicing | Splicing | 0.0006/- | 0.455:T |
| *CIZ1* | Het | chr9:130943046 | - | G | c.635dupC | p.E213Rfs*3 | Frameshift | -/- | - |
| *CIZ1* | Het | chr9:130947831 | G | A | c.583C>T | p.R195X | Stopgain | -/- | 0.503:T |
| *CIZ1* | Het | chr9:130952676 | C | A | c.218G>T | p.G73V | Missense | -/- | 0.710:D |
| *DRD2* | Het | chr11:113281460 | G | T | c.1321C>A | p.L441I | Missense | 0.00005798/- | 0.706:D |
| *DRD2* | Het | chr11:113283362 | G | A | c.1054C>T | p.R352W | Missense | 0/- | 0.720:D |
| *DRD2* | Het | chr11:113283439 | T | C | c.977A>G | p.E326G | Missense | -/- | 0.728:D |
| *DRD2* | Het | chr11:113286268 | C | T | c.598G>A | p.V200M | Missense | 0.0005/0.0006 | 0.882:D |
| *DRD2* | Het | chr11:113287657 | C | T | c.460G>A | p.V154I | Missense | 0/0 | 0.802:D |
| *DRD2* | Het | chr11:113295264 | T | C | c.110A>G | p.Y37C | Missense | 0/0 | 0.863:D |
| *EIF2AK2* | Het | chr2:37334495 | C | T | c.1577G>A | p.R526Q | Missense | -/- | 0.829:D |
| *EIF2AK2* | Het | chr2:37341888 | T | C | c.1363A>G | p.M455V | Missense | -/- | 0.714:D |
| *GNAL* | Het | chr18:11753825 | A | G | c.505A>G | p.T169A | Missense; Splicing | 0.0005/0.0006 | 0.901:D |
| *GNAL* | Het | chr18:11872275 | G | A | c.1040G>A | p.R347Q | Missense | 0/- | 0.883:D |
| *GNAL* | Het | chr18:11881090 | G | A | c.1333G>A | p.D445N | Missense | 0/0 | 0.704:D |
| *GNAO1* | Het | chr16:56362627 | C | T | c.388C>T | p.R130W | Missense | 0/- | 0.947:D |
| *GNAO1* | Het | chr16:56374810 | C | T | c.788C>T | p.T263M | Missense | 0/- | 0.960:D |
| *GNB1* | Het | chr1:1724687 | G | A | c.494C>T | p.T165M | Missense | 0/- | 0.877:D |
| *IRF2BPL* | Het | chr14:77491852 | C | T | c.2284G>A | p.E762K | Missense | -/- | 0.847:D |
| *IRF2BPL* | Het | chr14:77492022 | G | A | c.2114C>T | p.S705F | Missense | 0/- | 0.821:D |
| *IRF2BPL* | Het | chr14:77492271 | G | A | c.1865C>T | p.S622F | Missense | 0/0 | 0.767:D |
| *IRF2BPL* | Het | chr14:77492487 | G | A | c.1649C>T | p.P550L | Missense | 0/- | 0.711:D |
| *IRF2BPL* | Het | chr14:77494041 | T | G | c.95A>C | p.E32A | Missense | 0.00006015/- | 0.890:D |
| *IRF2BPL* | Het | chr14:77494060 | T | C | c.76A>G | p.M26V | Missense | -/- | 0.712:D |
| *KCNA1* | Het | chr12:5020870 | C | T | c.326C>T | p.P109L | Missense | -/- | 0.792:D |
| *KMT2B* | Het | chr19:36209200 | G | A | c.280G>A | p.G94R | Missense | -/- | 0.753:D |
| *KMT2B* | Het | chr19:36210742 | C | T | c.493C>T | p.R165C | Missense | 0/- | 0.763:D |
| *KMT2B* | Het | chr19:36211108 | C | T | c.859C>T | p.R287C | Missense | 0.0002/- | 0.703:D |
| *KMT2B* | Het | chr19:36211132 | C | T | c.883C>T | p.R295C | Missense | 0.0002/0.0006 | 0.763:D |
| *KMT2B* | Het | chr19:36211198 | G | A | c.949G>A | p.G317R | Missense | -/- | 0.713:D |
| *KMT2B* | Het | chr19:36212600 | C | T | c.2351C>T | p.P784L | Missense | 0/- | 0.775:D |
| *KMT2B* | Het | chr19:36213572 | C | T | c.2674C>T | p.R892C | Missense | 0.0001/- | 0.895:D |
| *KMT2B* | Het | chr19:36216639 | G | A | c.3805G>A | p.E1269K | Missense | 0/- | 0.866:D |
| *KMT2B* | Het | chr19:36216652 | A | G | c.3818A>G | p.H1273R | Missense | 0/- | 0.837:D |
| *KMT2B* | Het | chr19:36216694 | G | C | c.3860G>C | p.R1287P | Missense | -/- | 0.809:D |
| *KMT2B* | Het | chr19:36218387 | C | T | c.4166C>T | p.T1389I | Missense | -/- | 0.859:D |
| *KMT2B* | Het | chr19:36218396 | C | T | c.4175C>T | p.P1392L | Missense | 0/0 | 0.809:D |
| *KMT2B* | Het | chr19:36218848 | C | T | c.4459C>T | p.R1487C | Missense | 0/- | 0.775:D |
| *KMT2B* | Het | chr19:36219676 | G | A | c.4573G>A | p.G1525R | Missense; Splicing | 0/- | 0.832:D |
| *KMT2B* | Het | chr19:36221292 | A | G | c.5126A>G | p.D1709G | Missense | 0.00005798/- | 0.900:D |
| *KMT2B* | Het | chr19:36221448 | G | A | c.5207G>A | p.R1736H | Missense | 0.0005/- | 0.713:D |
| *KMT2B* | Het | chr19:36223155 | G | T | c.5705G>T | p.G1902V | Missense | -/- | 0.827:D |
| *KMT2B* | Het | chr19:36223269 | G | C | c.5819G>C | p.R1940T | Missense | -/- | 0.715:D |
| *KMT2B* | Het | chr19:36223619 | C | T | c.6169C>T | p.R2057C | Missense | 0.0001/- | 0.824:D |
| *KMT2B* | Het | chr19:36224298 | C | T | c.6848C>T | p.T2283I | Missense | -/- | 0.766:D |
| *NR4A2* | Het | chr2:157182300 | G | A | c.1753C>T | p.P585S | Missense | -/- | 0.828:D |
| *NR4A2* | Het | chr2:157182423 | C | A | c.1630G>T | p.G544W | Missense | 0.00005799/- | 0.810:D |
| *NR4A2* | Het | chr2:157186113 | C | G | c.586G>C | p.G196R | Missense | 0/- | 0.845:D |
| *NR4A2* | Het | chr2:157186142 | G | A | c.557C>T | p.P186L | Missense | 0/- | 0.820:D |
| *PNKD* | Het | chr2:219135295 | C | T | c.37C>T | p.R13W | Missense | 0/0.0006 | 0.762:D |
| *PNKD* | Het | chr2:219135296 | G | - | c.38delG | p.A15Rfs*57 | Frameshift | 0/- | - |
| *PNKD* | Het | chr2:219204522 | C | T | c.253C>T | p.R85C | Missense | 0/- | 0.821:D |
| *PNKD* | Het | chr2:219205501 | C | A | c.516C>A | p.H172Q | Missense | 0.00005798/- | 0.997:D |
| *PNKD* | Het | chr2:219205507 | C | A | c.522C>A | p.H174Q | Missense | -/- | 0.997:D |
| *PNKD* | Het | chr2:219206750 | C | T | c.664C>T | p.R222W | Missense | 0.00005798/0 | 0.792:D |
| *PNKD* | Het | chr2:219206765 | C | T | c.679C>T | p.P227S | Missense | -/- | 0.995:D |
| *PNKD* | Het | chr2:219208226 | G | A | c.785G>A | p.R262Q | Missense | 0/0 | 0.986:D |
| *PNKD* | Het | chr2:219208272 | TG | - | c.831_832del | p.V278Afs*5 | Frameshift | -/0.0006 | - |
| *PNKD* | Het | chr2:219209648 | C | T | c.1102C>T | p.R368W | Missense | 0.0008/- | 0.920:D |
| *PRRT2* | Het | chr16:29824980 | CA | - | c.605_606del | p.P203Tfs*20 | Frameshift | -/- | - |
| *PRRT2* | Het | chr16:29825849 | - | C | c.1076dupC | p.A361Cfs*33 | Frameshift | -/- | - |
| *PRRT2* | Het | chr16:29825915 | C | - | c.1141delC | p.V383Sfs*47 | Frameshift | 0.0004/0 | - |
| *RHOBTB2* | Het | chr8:22862050 | G | A | c.124G>A | p.A42T | Missense | 0/0 | 0.802:D |
| *RHOBTB2* | Het | chr8:22864801 | G | C | c.1064G>C | p.C355S | Missense | -/- | 0.730:D |
| *RHOBTB2* | Het | chr8:22865248 | G | C | c.1511G>C | p.G504A | Missense | 0.0001/- | 0.827:D |
| *RHOBTB2* | Het | chr8:22868106 | A | G | c.1697A>G | p.Y566C | Missense | -/- | 0.992:D |
| *SCN8A* | Het | chr12:52056684 | G | T | c.83G>T | p.R28L | Missense | -/- | 0.881:D |
| *SCN8A* | Het | chr12:52056734 | C | T | c.133C>T | p.R45W | Missense | 0/0 | 0.895:D |
| *SCN8A* | Het | chr12:52100452 | C | T | c.1588C>T | p.R530W | Missense | 0.0002/- | 0.799:D |
| *SCN8A* | Het | chr12:52100468 | G | A | c.1604G>A | p.R535K | Missense | -/- | 0.812:D |
| *SCN8A* | Het | chr12:52139748 | A | G | c.2060A>G | p.Q687R | Missense | 0.00005817/0.0006 | 0.904:D |
| *SCN8A* | Het | chr12:52145288 | A | G | c.2281A>G | p.I761V | Missense | 0.0006/- | 0.852:D |
| *SCN8A* | Het | chr12:52159773 | A | G | c.2863A>G | p.I955V | Missense | -/- | 0.701:D |
| *SCN8A* | Het | chr12:52188354 | C | T | c.4724C>T | p.A1575V | Missense | 0.00005801/- | 0.967:D |
| *SCN8A* | Het | chr12:52200720 | A | G | c.5450A>G | p.H1817R | Missense | -/- | 0.824:D |
| *SGCE* | Het | chr7:94228099 | A | G | c.1214T>C | p.M405T | Missense | -/- | 0.908:D |
| *SGCE* | Het | chr7:94228124 | A | C | c.1189T>G | p.Y397D | Missense | -/- | 0.911:D |
| *SGCE* | Het | chr7:94228225 | C | T | c.1088G>A | p.R363Q | Missense | 0/- | 0.751:D |
| *SGCE* | Het | chr7:94230090 | T | A | c.905A>T | p.K302I | Missense | 0.00005827/0 | 0.744:D |
| *SGCE* | Het | chr7:94230150 | A | G | c.845T>C | p.V282A | Missense | -/- | 0.844:D |
| *SGCE* | Het | chr7:94232692 | T | A | c.735A>T | p.Q245H | Missense | -/- | 0.791:D |
| *SGCE* | Het | chr7:94248176 | C | T | c.556G>A | p.A186T | Missense | 0.0003/0 | 0.940:D |
| *SGCE* | Het | chr7:94248254 | A | G | c.478T>C | p.Y160H | Missense | -/- | 0.944:D |
| *SGCE* | Het | chr7:94252690 | C | T | c.410G>A | p.R137H | Missense | 0.0001/0 | 0.864:D |
| *SGCE* | Het | chr7:94252693 | C | T | c.407G>A | p.R136K | Missense | -/0 | 0.797:D |
| *SGCE* | Het | chr7:94259120 | C | T | c.143G>A | p.R48Q | Missense | 0/- | 0.764:D |
| *SGCE* | Het | chr7:94285344 | G | A | c.67C>T | p.R23C | Missense | -/- | 0.746:D |
| *SPR* | Het | chr2:73118506 | C | T | c.626C>T | p.A209V | Missense | 0.00005798/- | 0.822:D |
| *THAP1* | Het | chr8:42693124 | A | G | c.623T>C | p.I208T | Missense | -/- | 0.717:D |
| *THAP1* | Het | chr8:42693226 | T | C | c.521A>G | p.E174G | Missense | 0.0001/- | 0.895:D |
| *THAP1* | Het | chr8:42693320 | T | C | c.427A>G | p.M143V | Missense | 0.0005/0.0006 | 0.775:D |
| *TOR1A* | Het | chr9:132584985 | C | T | c.319G>A | p.G107S | Missense | 0/- | 0.983:D |
| *TOR1A* | Het | chr9:132586362 | C | T | c.3G>A | p.M1I | Missense | -/- | 0.816:D |
| *TUBB4A* | Het | chr19:6495788 | C | G | c.722G>C | p.R241P | Missense | -/- | 0.971:D |
| *VPS16* | Het | chr20:2840397 | T | C | c.86T>C | p.L29P | Missense | 0.0004/0.0006 | 0.828:D |
| *VPS16* | Het | chr20:2841677 | A | G | c.692A>G | p.Y231C | Missense | 0.0003/- | 0.725:D |
| *VPS16* | Het | chr20:2843241 | C | T | c.1088C>T | p.A363V | Missense | 0/0 | 0.705:D |
| *VPS16* | Het | chr20:2843513 | T | C | c.1264T>C | p.C422R | Missense | -/- | 0.898:D |
| *VPS16* | Het | chr20:2843565 | C | T | c.1316C>T | p.P439L | Missense | 0/0 | 0.856:D |
| *VPS16* | Het | chr20:2844030 | T | C | c.1462T>C | p.W488R | Missense | -/- | 0.986:D |
| *VPS16* | Het | chr20:2844679 | G | T | c.1561G>T | p.D521Y | Missense | 0/- | 0.719:D |
| *VPS16* | Het | chr20:2844710 | G | A | c.1592G>A | p.R531H | Missense | -/- | 0.968:D |
| *VPS16* | Het | chr20:2844912 | G | A | c.1699G>A | p.E567K | Missense | -/0.0006 | 0.725:D |
| *VPS16* | Het | chr20:2845103 | C | G | c.1813C>G | p.R605G | Missense | 0.00005798/- | 0.789:D |
| *VPS16* | Het | chr20:2845230 | T | C | c.1856T>C | p.L619P | Missense | -/- | 0.964:D |
| *VPS16* | Het | chr20:2845261 | G | T | c.1887G>T | p.L629F | Missense | -/- | 0.728:D |
| *VPS16* | Het | chr20:2845287 | A | G | c.1913A>G | p.Y638C | Missense | -/0.0006 | 0.826:D |
| *VPS16* | Het | chr20:2846897 | G | A | c.2311G>A | p.E771K | Missense | 0.0001/- | 0.927:D |
| *VPS16* | Het | chr20:2846921 | G | A | c.2335G>A | p.V779M | Missense | 0/- | 0.704:D |

^a^ Minor allele frequency in East Asian population of gnomAD exome database and genome database. “-” means the variant was not found in the gnomAD exome or genome database. Hom: Homozygous; Het: Heterozygous; Deleterious: the combination of damaging missense (missense variants with ReVe ≥ 0.7) and loss of function variants were defined as “deleterious”.

**Supplementary Table 5.** The clinical phenotype of patients with Parkinson’s disease harboring monoallelic potentially pathogenic variants of dominant dystonia-related genes.

| **Case** | **LOPD-1445** | **EOPD-0001** | **EOPD-0190** | **EOPD-1469** | **LOPD-0694** |
| --- | --- | --- | --- | --- | --- |
| DYT gene | *ADCY5* | *ANO3* | *ANO3* | *ANO3* | *SLC2A1* |
| Sex | M | M | M | M | M |
| Age at onset(yrs) | 59 | 42 | 42 | 49 | 77 |
| Age at examination(yrs) | 74 | 53 | 45 | 52 | 80 |
| Disease duration(yrs) | 15 | 11 | 3 | 3 | 3 |
| Hoehn-Yahr stage | 4 | 3 | 2.5 | 2 | 2 |
| Bradykinesia | + | + | + | + | + |
| Resting tremor | + | + | – | + | + |
| Rigidity | + | + | + | + | + |
| Postural instability | + | + | – | – | – |
| UPDRS-III | 58 | 42 | 30 | 22 | 25 |
| Motor subtype | PIGD | TD | PIGD | TD | PIGD |
| Hyposmia | + | + | – | – | + |
| Depression | + | + | – | – | + |
| Urinary urgency | + | – | – | – | + |
| Constipation | + | – | – | – | + |
| Cognitive decline | – | – | – | – | – |
| MMSE | 25 | 25 | 29 | 30 | 26 |
| Sleep disturbance | – | – | – | – | + |
| RBD | + | – | – | – | – |
| Freezing gait | + | – | + | – | – |
| Response to levodopa | good | good | good | good | good |
| Wearing off | + | + | – | – | – |
| Dyskinesia | – | + | – | – | – |
| PD diagnosis * | EstPD | EstPD | EstPD | EstPD | EstPD |

* PD diagnosis means cases were classified in accordance with the Movement Disorder Society clinical diagnostic criteria for Parkinson’s disease. –: absent; +: present; yrs: years; PIGD: postural instability and gait disorders; TD: Tremor dominant; PD: Parkinson’s disease; EstPD: Established PD.

**Supplementary Table 6.** Burden analyses of each dystonia-related gene in Parkinson’s disease.

| **Gene** | **Cohort** | **Patient subgroup** | **Variant**  **subgroup ^a^** | **MAF < 0.001** | |  | **MAF < 0.01** | |
| --- | --- | --- | --- | --- | --- | --- | --- | --- |
|  |  |  |  | **Variants included** | **SKAT-O p value** |  | **Variants included** | **SKAT-O p value** |
| *ADCY5* | Cohort 1 | sEOPD | Missense | 20 | 0.535653728 |  | 23 | 0.048647705 |
| *ADCY5* | Cohort 1 | sEOPD | Dmis | 7 | 0.677541516 |  | 7 | 0.677541516 |
| *ADCY5* | Cohort 1 | sEOPD | Deleterious | 7 | 0.677541516 |  | 7 | 0.677541516 |
| *ADCY5* | Cohort 2 | sLOPD | Missense | 30 | 0.389092621 |  | 31 | 0.681585532 |
| *ADCY5* | Cohort 2 | sLOPD | Dmis | 10 | 0.759601718 |  | 10 | 0.759601718 |
| *ADCY5* | Cohort 2 | sLOPD | Deleterious | 10 | 0.759601718 |  | 10 | 0.759601718 |
| *ANO3* | Cohort 1 | sEOPD | LoF | 2 | 0.550036296 |  | 2 | 0.550036296 |
| *ANO3* | Cohort 1 | sEOPD | Missense | 24 | 0.570369503 |  | 24 | 0.570369503 |
| *ANO3* | Cohort 1 | sEOPD | Dmis | 8 | 0.818309516 |  | 8 | 0.818309516 |
| *ANO3* | Cohort 1 | sEOPD | Deleterious | 10 | 0.843334507 |  | 10 | 0.843334507 |
| *ANO3* | Cohort 2 | sLOPD | Missense | 23 | 0.932168905 |  | 24 | 0.90716419 |
| *ANO3* | Cohort 2 | sLOPD | Dmis | 8 | 0.574554945 |  | 8 | 0.574554945 |
| *ANO3* | Cohort 2 | sLOPD | Deleterious | 8 | 0.574554945 |  | 8 | 0.574554945 |
| *ATP1A3* | Cohort 1 | sEOPD | Missense | 13 | 0.767868026 |  | 13 | 0.767868026 |
| *ATP1A3* | Cohort 1 | sEOPD | Dmis | 4 | 0.357850167 |  | 4 | 0.357850167 |
| *ATP1A3* | Cohort 1 | sEOPD | Deleterious | 4 | 0.357850167 |  | 4 | 0.357850167 |
| *ATP1A3* | Cohort 2 | sLOPD | Missense | 7 | 0.191889431 |  | 7 | 0.191889431 |
| *CACNA1A* | Cohort 1 | sEOPD | LoF | 4 | 0.681327167 |  | 4 | 0.681327167 |
| *CACNA1A* | Cohort 1 | sEOPD | Missense | 71 | 0.141908859 |  | 77 | 0.049771177 |
| *CACNA1A* | Cohort 1 | sEOPD | Dmis | 11 | 0.113702214 |  | 11 | 0.113702214 |
| *CACNA1A* | Cohort 1 | sEOPD | Deleterious | 15 | 0.61209625 |  | 15 | 0.61209625 |
| *CACNA1A* | Cohort 2 | sLOPD | Missense | 49 | 0.431506481 |  | 58 | 0.505871463 |
| *CACNA1A* | Cohort 2 | sLOPD | Dmis | 8 | 0.736244408 |  | 9 | 0.67549678 |
| *CACNA1A* | Cohort 2 | sLOPD | Deleterious | 8 | 0.736244408 |  | 9 | 0.67549678 |
| *CACNA1B* | Cohort 1 | sEOPD | LoF | 1 | 0.492543899 |  | 2 | 0.243809877 |
| *CACNA1B* | Cohort 1 | sEOPD | Missense | 75 | 0.166224992 |  | 87 | 0.034354134 |
| *CACNA1B* | Cohort 1 | sEOPD | Dmis | 33 | 0.415052209 |  | 38 | 0.341025428 |
| *CACNA1B* | Cohort 1 | sEOPD | Deleterious | 34 | 0.440278924 |  | 40 | 0.301717795 |
| *CACNA1B* | Cohort 2 | sLOPD | LoF | 1 | 0.416145295 |  | 1 | 0.416145295 |
| *CACNA1B* | Cohort 2 | sLOPD | Missense | 48 | 0.55933068 |  | 58 | 0.236760067 |
| *CACNA1B* | Cohort 2 | sLOPD | Dmis | 15 | 0.711166357 |  | 19 | 0.393558293 |
| *CACNA1B* | Cohort 2 | sLOPD | Deleterious | 16 | 0.607228427 |  | 20 | 0.324999966 |
| *CIZ1* | Cohort 1 | sEOPD | LoF | 4 | 0.738633464 |  | 4 | 0.738633464 |
| *CIZ1* | Cohort 1 | sEOPD | Missense | 32 | 0.847996031 |  | 36 | 0.857791739 |
| *CIZ1* | Cohort 1 | sEOPD | Dmis | 3 | 0.721034415 |  | 3 | 0.721034415 |
| *CIZ1* | Cohort 1 | sEOPD | Deleterious | 7 | 0.597348301 |  | 7 | 0.597348301 |
| *CIZ1* | Cohort 2 | sLOPD | LoF | 2 | 1 |  | 2 | 1 |
| *CIZ1* | Cohort 2 | sLOPD | Missense | 30 | 0.794816116 |  | 34 | 0.324690829 |
| *CIZ1* | Cohort 2 | sLOPD | Deleterious | 2 | 1 |  | 2 | 1 |
| *COL6A3* | Cohort 1 | sEOPD | LoF | 3 | 0.298360255 |  | 3 | 0.298360255 |
| *COL6A3* | Cohort 1 | sEOPD | Missense | 163 | 0.782822995 |  | 193 | 0.831815787 |
| *COL6A3* | Cohort 1 | sEOPD | Dmis | 66 | 1 |  | 77 | 0.631112064 |
| *COL6A3* | Cohort 1 | sEOPD | Deleterious | 69 | 1 |  | 80 | 0.620722032 |
| *COL6A3* | Cohort 2 | sLOPD | Missense | 125 | 0.271015454 |  | 154 | 0.069437542 |
| *COL6A3* | Cohort 2 | sLOPD | Dmis | 53 | 0.121206 |  | 63 | 0.034551114 |
| *COL6A3* | Cohort 2 | sLOPD | Deleterious | 53 | 0.121206 |  | 63 | 0.034551114 |
| *COQ8A* | Cohort 1 | sEOPD | LoF | 6 | 0.408382425 |  | 6 | 0.408382425 |
| *COQ8A* | Cohort 1 | sEOPD | Missense | 44 | 0.734376947 |  | 53 | 0.883302764 |
| *COQ8A* | Cohort 1 | sEOPD | Dmis | 17 | 0.698224741 |  | 19 | 1 |
| *COQ8A* | Cohort 1 | sEOPD | Deleterious | 23 | 0.799789732 |  | 25 | 0.827967972 |
| *COQ8A* | Cohort 2 | sLOPD | LoF | 2 | 1 |  | 2 | 1 |
| *COQ8A* | Cohort 2 | sLOPD | Missense | 27 | 0.913273764 |  | 34 | 0.571363204 |
| *COQ8A* | Cohort 2 | sLOPD | Dmis | 10 | 0.504298191 |  | 11 | 0.34542555 |
| *COQ8A* | Cohort 2 | sLOPD | Deleterious | 12 | 0.737618364 |  | 13 | 0.424479636 |
| *CP* | Cohort 1 | sEOPD | LoF | 2 | 0.350509942 |  | 2 | 0.350509942 |
| *CP* | Cohort 1 | sEOPD | Missense | 35 | 0.25444571 |  | 43 | 0.133669007 |
| *CP* | Cohort 1 | sEOPD | Dmis | 15 | 0.359142317 |  | 16 | 0.06449706 |
| *CP* | Cohort 1 | sEOPD | Deleterious | 17 | 0.343200854 |  | 18 | 0.064947568 |
| *CP* | Cohort 2 | sLOPD | LoF | 2 | 0.449897033 |  | 2 | 0.449897033 |
| *CP* | Cohort 2 | sLOPD | Missense | 32 | 0.181816722 |  | 40 | 0.285781919 |
| *CP* | Cohort 2 | sLOPD | Dmis | 15 | 0.245051834 |  | 17 | 0.379163874 |
| *CP* | Cohort 2 | sLOPD | Deleterious | 17 | 0.298679308 |  | 19 | 0.423232228 |
| *DDC* | Cohort 1 | sEOPD | LoF | 2 | 0.578242942 |  | 2 | 0.578242942 |
| *DDC* | Cohort 1 | sEOPD | Missense | 21 | 0.187641134 |  | 22 | 0.08339731 |
| *DDC* | Cohort 1 | sEOPD | Dmis | 9 | 0.090894641 |  | 10 | 0.062274079 |
| *DDC* | Cohort 1 | sEOPD | Deleterious | 11 | 0.102970836 |  | 12 | 0.064508808 |
| *DDC* | Cohort 2 | sLOPD | Missense | 19 | 0.674913803 |  | 20 | 0.707308873 |
| *DDC* | Cohort 2 | sLOPD | Dmis | 9 | 0.615365047 |  | 10 | 1 |
| *DDC* | Cohort 2 | sLOPD | Deleterious | 9 | 0.615365047 |  | 10 | 1 |
| *DRD2* | Cohort 1 | sEOPD | Missense | 7 | 0.299493415 |  | 8 | 0.358583091 |
| *DRD2* | Cohort 1 | sEOPD | Dmis | 4 | 0.289599538 |  | 5 | 0.362475424 |
| *DRD2* | Cohort 1 | sEOPD | Deleterious | 4 | 0.289599538 |  | 5 | 0.362475424 |
| *DRD2* | Cohort 2 | sLOPD | Missense | 8 | 0.335256902 |  | 9 | 0.489184034 |
| *DRD2* | Cohort 2 | sLOPD | Dmis | 3 | 0.509934183 |  | 4 | 0.639082174 |
| *DRD2* | Cohort 2 | sLOPD | Deleterious | 3 | 0.509934183 |  | 4 | 0.639082174 |
| *EIF2AK2* | Cohort 1 | sEOPD | Missense | 8 | 1 |  | 10 | 0.766307716 |
| *EIF2AK2* | Cohort 1 | sEOPD | Dmis | 1 | 0.294039855 |  | 1 | 0.294039855 |
| *EIF2AK2* | Cohort 1 | sEOPD | Deleterious | 1 | 0.294039855 |  | 1 | 0.294039855 |
| *EIF2AK2* | Cohort 2 | sLOPD | Missense | 3 | 0.272041057 |  | 5 | 0.696098462 |
| *EIF2AK2* | Cohort 2 | sLOPD | Dmis | 1 | 0.474704851 |  | 1 | 0.474704851 |
| *EIF2AK2* | Cohort 2 | sLOPD | Deleterious | 1 | 0.474704851 |  | 1 | 0.474704851 |
| *GNAL* | Cohort 1 | sEOPD | Missense | 13 | 0.854252359 |  | 13 | 0.854252359 |
| *GNAL* | Cohort 1 | sEOPD | Dmis | 1 | 0.524907976 |  | 1 | 0.524907976 |
| *GNAL* | Cohort 1 | sEOPD | Deleterious | 1 | 0.524907976 |  | 1 | 0.524907976 |
| *GNAL* | Cohort 2 | sLOPD | Missense | 7 | 0.359457236 |  | 7 | 0.359457236 |
| *GNAL* | Cohort 2 | sLOPD | Dmis | 1 | 0.255830272 |  | 1 | 0.255830272 |
| *GNAL* | Cohort 2 | sLOPD | Deleterious | 1 | 0.255830272 |  | 1 | 0.255830272 |
| *GNAO1* | Cohort 1 | sEOPD | Missense | 7 | 0.79013796 |  | 8 | 0.699332231 |
| *GNAO1* | Cohort 1 | sEOPD | Dmis | 2 | 0.49922195 |  | 3 | 0.815822007 |
| *GNAO1* | Cohort 1 | sEOPD | Deleterious | 2 | 0.49922195 |  | 3 | 0.815822007 |
| *GNAO1* | Cohort 2 | sLOPD | Missense | 4 | 0.659995989 |  | 5 | 0.851016815 |
| *GNAO1* | Cohort 2 | sLOPD | Dmis | 3 | 0.439446872 |  | 4 | 0.542026773 |
| *GNAO1* | Cohort 2 | sLOPD | Deleterious | 3 | 0.439446872 |  | 4 | 0.542026773 |
| *GNB1* | Cohort 1 | sEOPD | Missense | 5 | 0.788444381 |  | 5 | 0.788444381 |
| *GNB1* | Cohort 1 | sEOPD | Dmis | 1 | 0.44549371 |  | 1 | 0.44549371 |
| *GNB1* | Cohort 1 | sEOPD | Deleterious | 1 | 0.44549371 |  | 1 | 0.44549371 |
| *GNB1* | Cohort 2 | sLOPD | Missense | 1 | 0.368791248 |  | 1 | 0.368791248 |
| *IRF2BPL* | Cohort 1 | sEOPD | LoF | 2 | 0.152204207 |  | 2 | 0.152204207 |
| *IRF2BPL* | Cohort 1 | sEOPD | Missense | 25 | 0.600251516 |  | 30 | 0.794549582 |
| *IRF2BPL* | Cohort 1 | sEOPD | Dmis | 3 | 0.415323041 |  | 3 | 0.415323041 |
| *IRF2BPL* | Cohort 1 | sEOPD | Deleterious | 5 | 0.249292973 |  | 5 | 0.249292973 |
| *IRF2BPL* | Cohort 2 | sLOPD | Missense | 26 | 0.748567856 |  | 29 | 0.712623538 |
| *IRF2BPL* | Cohort 2 | sLOPD | Dmis | 4 | 0.106639922 |  | 4 | 0.106639922 |
| *IRF2BPL* | Cohort 2 | sLOPD | Deleterious | 4 | 0.106639922 |  | 4 | 0.106639922 |
| *KCNA1* | Cohort 1 | sEOPD | Missense | 10 | 0.372393812 |  | 10 | 0.372393812 |
| *KCNA1* | Cohort 1 | sEOPD | Dmis | 3 | 0.436696076 |  | 3 | 0.436696076 |
| *KCNA1* | Cohort 1 | sEOPD | Deleterious | 3 | 0.436696076 |  | 3 | 0.436696076 |
| *KCNA1* | Cohort 2 | sLOPD | Missense | 5 | 0.182439651 |  | 5 | 0.182439651 |
| *KCNA1* | Cohort 2 | sLOPD | Dmis | 1 | 0.22726741 |  | 1 | 0.22726741 |
| *KCNA1* | Cohort 2 | sLOPD | Deleterious | 1 | 0.22726741 |  | 1 | 0.22726741 |
| *KCTD17* | Cohort 1 | sEOPD | LoF | 1 | 0.402912324 |  | 1 | 0.402912324 |
| *KCTD17* | Cohort 1 | sEOPD | Missense | 12 | 0.730841728 |  | 13 | 0.612574653 |
| *KCTD17* | Cohort 1 | sEOPD | Deleterious | 1 | 0.402912324 |  | 1 | 0.402912324 |
| *KCTD17* | Cohort 2 | sLOPD | LoF | 1 | 0.437642633 |  | 1 | 0.437642633 |
| *KCTD17* | Cohort 2 | sLOPD | Missense | 8 | 0.49521217 |  | 9 | 0.455954217 |
| *KCTD17* | Cohort 2 | sLOPD | Deleterious | 1 | 0.437642633 |  | 1 | 0.437642633 |
| *KMT2B* | Cohort 1 | sEOPD | Missense | 103 | 0.724856043 |  | 114 | 0.919020892 |
| *KMT2B* | Cohort 1 | sEOPD | Dmis | 20 | 0.570997467 |  | 20 | 0.570997467 |
| *KMT2B* | Cohort 1 | sEOPD | Deleterious | 20 | 0.570997467 |  | 20 | 0.570997467 |
| *KMT2B* | Cohort 2 | sLOPD | Missense | 75 | 0.422961278 |  | 83 | 0.792648802 |
| *KMT2B* | Cohort 2 | sLOPD | Dmis | 13 | 0.407177725 |  | 13 | 0.407177725 |
| *KMT2B* | Cohort 2 | sLOPD | Deleterious | 13 | 0.407177725 |  | 13 | 0.407177725 |
| *MECR* | Cohort 1 | sEOPD | LoF | 1 | 0.646052368 |  | 1 | 0.646052368 |
| *MECR* | Cohort 1 | sEOPD | Missense | 23 | 0.349637083 |  | 23 | 0.349637083 |
| *MECR* | Cohort 1 | sEOPD | Dmis | 4 | 0.082796427 |  | 4 | 0.082796427 |
| *MECR* | Cohort 1 | sEOPD | Deleterious | 5 | 0.156614102 |  | 5 | 0.156614102 |
| *MECR* | Cohort 2 | sLOPD | LoF | 2 | 0.548037739 |  | 2 | 0.548037739 |
| *MECR* | Cohort 2 | sLOPD | Missense | 15 | 0.74451621 |  | 15 | 0.74451621 |
| *MECR* | Cohort 2 | sLOPD | Dmis | 3 | 0.280248731 |  | 3 | 0.280248731 |
| *MECR* | Cohort 2 | sLOPD | Deleterious | 5 | 0.685689826 |  | 5 | 0.685689826 |
| *MED27* | Cohort 1 | sEOPD | LoF | 1 | 0.306891894 |  | 1 | 0.306891894 |
| *MED27* | Cohort 1 | sEOPD | Missense | 4 | 0.247348127 |  | 4 | 0.247348127 |
| *MED27* | Cohort 1 | sEOPD | Dmis | 1 | 0.416345144 |  | 1 | 0.416345144 |
| *MED27* | Cohort 1 | sEOPD | Deleterious | 2 | 0.27252809 |  | 2 | 0.27252809 |
| *MED27* | Cohort 2 | sLOPD | Missense | 5 | 0.837846169 |  | 6 | 0.914681484 |
| *MED27* | Cohort 2 | sLOPD | Dmis | 1 | 0.376422088 |  | 1 | 0.376422088 |
| *MED27* | Cohort 2 | sLOPD | Deleterious | 1 | 0.376422088 |  | 1 | 0.376422088 |
| *NR4A2* | Cohort 1 | sEOPD | Missense | 11 | 0.005765831 |  | 14 | 0.365047978 |
| *NR4A2* | Cohort 1 | sEOPD | Dmis | 5 | 0.027247032 |  | 7 | 0.352663653 |
| *NR4A2* | Cohort 1 | sEOPD | Deleterious | 5 | 0.027247032 |  | 7 | 0.352663653 |
| *NR4A2* | Cohort 2 | sLOPD | Missense | 7 | 0.741091682 |  | 10 | 0.898658513 |
| *NR4A2* | Cohort 2 | sLOPD | Dmis | 4 | 0.561576372 |  | 7 | 0.851152704 |
| *NR4A2* | Cohort 2 | sLOPD | Deleterious | 4 | 0.561576372 |  | 7 | 0.851152704 |
| *PNKD* | Cohort 1 | sEOPD | LoF | 4 | 0.800006712 |  | 4 | 0.800006712 |
| *PNKD* | Cohort 1 | sEOPD | Missense | 24 | 0.111150019 |  | 27 | 0.085538763 |
| *PNKD* | Cohort 1 | sEOPD | Dmis | 13 | 0.387312189 |  | 14 | 0.494079454 |
| *PNKD* | Cohort 1 | sEOPD | Deleterious | 17 | 0.33927283 |  | 18 | 0.431309417 |
| *PNKD* | Cohort 2 | sLOPD | LoF | 1 | 0.239370698 |  | 1 | 0.239370698 |
| *PNKD* | Cohort 2 | sLOPD | Missense | 22 | 0.213409337 |  | 25 | 0.145311692 |
| *PNKD* | Cohort 2 | sLOPD | Dmis | 9 | 0.131838931 |  | 10 | 0.427011474 |
| *PNKD* | Cohort 2 | sLOPD | Deleterious | 10 | 0.128757678 |  | 11 | 0.420208469 |
| *PRKRA* | Cohort 1 | sEOPD | Missense | 7 | 1 |  | 8 | 1 |
| *PRKRA* | Cohort 1 | sEOPD | Dmis | 1 | 0.513924492 |  | 2 | 0.727856302 |
| *PRKRA* | Cohort 1 | sEOPD | Deleterious | 1 | 0.513924492 |  | 2 | 0.727856302 |
| *PRKRA* | Cohort 2 | sLOPD | Missense | 7 | 0.386184723 |  | 8 | 0.396127032 |
| *PRKRA* | Cohort 2 | sLOPD | Dmis | 1 | 0.52344945 |  | 2 | 0.406472235 |
| *PRKRA* | Cohort 2 | sLOPD | Deleterious | 1 | 0.52344945 |  | 2 | 0.406472235 |
| *PRRT2* | Cohort 1 | sEOPD | LoF | 1 | 0.403182303 |  | 2 | 0.573481448 |
| *PRRT2* | Cohort 1 | sEOPD | Missense | 24 | 0.805400835 |  | 28 | 0.597002648 |
| *PRRT2* | Cohort 1 | sEOPD | Dmis | 1 | 0.146382795 |  | 1 | 0.146382795 |
| *PRRT2* | Cohort 1 | sEOPD | Deleterious | 2 | 0.443929714 |  | 3 | 0.543987878 |
| *PRRT2* | Cohort 2 | sLOPD | Missense | 12 | 0.461667123 |  | 14 | 0.548338981 |
| *PTS* | Cohort 1 | sEOPD | LoF | 4 | 0.586393991 |  | 4 | 0.586393991 |
| *PTS* | Cohort 1 | sEOPD | Missense | 3 | 0.360297765 |  | 5 | 0.198064976 |
| *PTS* | Cohort 1 | sEOPD | Dmis | 1 | 0.230379715 |  | 2 | 0.368104813 |
| *PTS* | Cohort 1 | sEOPD | Deleterious | 5 | 0.36695671 |  | 6 | 0.679564562 |
| *PTS* | Cohort 2 | sLOPD | LoF | 1 | 0.391209153 |  | 1 | 0.391209153 |
| *PTS* | Cohort 2 | sLOPD | Missense | 5 | 0.297839913 |  | 7 | 0.257739587 |
| *PTS* | Cohort 2 | sLOPD | Dmis | 4 | 0.412435917 |  | 5 | 0.312333697 |
| *PTS* | Cohort 2 | sLOPD | Deleterious | 5 | 0.471761001 |  | 6 | 0.210486652 |
| *QDPR* | Cohort 1 | sEOPD | LoF | 1 | 0.279696003 |  | 1 | 0.279696003 |
| *QDPR* | Cohort 1 | sEOPD | Missense | 5 | 0.31332558 |  | 5 | 0.31332558 |
| *QDPR* | Cohort 1 | sEOPD | Dmis | 2 | 0.46382264 |  | 2 | 0.46382264 |
| *QDPR* | Cohort 1 | sEOPD | Deleterious | 3 | 0.261247621 |  | 3 | 0.261247621 |
| *QDPR* | Cohort 2 | sLOPD | LoF | 1 | 0.498182491 |  | 1 | 0.498182491 |
| *QDPR* | Cohort 2 | sLOPD | Missense | 8 | 0.394322486 |  | 8 | 0.394322486 |
| *QDPR* | Cohort 2 | sLOPD | Dmis | 4 | 0.450997251 |  | 4 | 0.450997251 |
| *QDPR* | Cohort 2 | sLOPD | Deleterious | 5 | 0.546210497 |  | 5 | 0.546210497 |
| *RHOBTB2* | Cohort 1 | sEOPD | LoF | 2 | 0.216151644 |  | 2 | 0.216151644 |
| *RHOBTB2* | Cohort 1 | sEOPD | Missense | 25 | 1 |  | 27 | 1 |
| *RHOBTB2* | Cohort 1 | sEOPD | Dmis | 3 | 0.642604621 |  | 4 | 0.468548729 |
| *RHOBTB2* | Cohort 1 | sEOPD | Deleterious | 5 | 0.462528518 |  | 6 | 0.29399353 |
| *RHOBTB2* | Cohort 2 | sLOPD | Missense | 14 | 0.300423315 |  | 17 | 0.305424971 |
| *RHOBTB2* | Cohort 2 | sLOPD | Dmis | 3 | 0.543508154 |  | 4 | 0.44968378 |
| *RHOBTB2* | Cohort 2 | sLOPD | Deleterious | 3 | 0.543508154 |  | 4 | 0.44968378 |
| *SCN8A* | Cohort 1 | sEOPD | Missense | 17 | 0.900653525 |  | 17 | 0.900653525 |
| *SCN8A* | Cohort 1 | sEOPD | Dmis | 11 | 0.917200418 |  | 11 | 0.917200418 |
| *SCN8A* | Cohort 1 | sEOPD | Deleterious | 11 | 0.917200418 |  | 11 | 0.917200418 |
| *SCN8A* | Cohort 2 | sLOPD | Missense | 20 | 0.093133149 |  | 20 | 0.093133149 |
| *SCN8A* | Cohort 2 | sLOPD | Dmis | 10 | 0.142688705 |  | 10 | 0.142688705 |
| *SCN8A* | Cohort 2 | sLOPD | Deleterious | 10 | 0.142688705 |  | 10 | 0.142688705 |
| *SGCE* | Cohort 1 | sEOPD | LoF | 0 | - |  | 1 | 0.656485519 |
| *SGCE* | Cohort 1 | sEOPD | Missense | 24 | 0.099466156 |  | 25 | 0.170828902 |
| *SGCE* | Cohort 1 | sEOPD | Dmis | 15 | 1 |  | 16 | 1 |
| *SGCE* | Cohort 1 | sEOPD | Deleterious | 15 | 1 |  | 17 | 1 |
| *SGCE* | Cohort 2 | sLOPD | LoF | 1 | 0.142237184 |  | 2 | 0.39121492 |
| *SGCE* | Cohort 2 | sLOPD | Missense | 13 | 0.556496692 |  | 16 | 0.228192153 |
| *SGCE* | Cohort 2 | sLOPD | Dmis | 8 | 0.757283678 |  | 9 | 0.092727916 |
| *SGCE* | Cohort 2 | sLOPD | Deleterious | 9 | 0.662364224 |  | 11 | 0.150015894 |
| *SLC18A2* | Cohort 1 | sEOPD | Missense | 22 | 0.302264882 |  | 26 | 0.036076594 |
| *SLC18A2* | Cohort 1 | sEOPD | Dmis | 7 | 0.539418829 |  | 8 | 0.583307082 |
| *SLC18A2* | Cohort 1 | sEOPD | Deleterious | 7 | 0.539418829 |  | 8 | 0.583307082 |
| *SLC18A2* | Cohort 2 | sLOPD | Missense | 15 | 0.218619456 |  | 19 | 0.430469513 |
| *SLC18A2* | Cohort 2 | sLOPD | Dmis | 3 | 0.772086265 |  | 4 | 0.223409642 |
| *SLC18A2* | Cohort 2 | sLOPD | Deleterious | 3 | 0.772086265 |  | 4 | 0.223409642 |
| *SLC2A1* | Cohort 1 | sEOPD | Missense | 9 | 0.024511996 |  | 10 | 0.050890759 |
| *SLC2A1* | Cohort 1 | sEOPD | Dmis | 1 | 0.292992486 |  | 1 | 0.292992486 |
| *SLC2A1* | Cohort 1 | sEOPD | Deleterious | 1 | 0.292992486 |  | 1 | 0.292992486 |
| *SLC2A1* | Cohort 2 | sLOPD | Missense | 16 | 0.006501917 |  | 17 | 0.014588115 |
| *SLC2A1* | Cohort 2 | sLOPD | Dmis | 1 | 0.352406112 |  | 1 | 0.352406112 |
| *SLC2A1* | Cohort 2 | sLOPD | Deleterious | 1 | 0.352406112 |  | 1 | 0.352406112 |
| *SLC30A10* | Cohort 1 | sEOPD | Missense | 19 | 0.382472607 |  | 21 | 0.776269411 |
| *SLC30A10* | Cohort 1 | sEOPD | Dmis | 4 | 0.809766713 |  | 4 | 0.809766713 |
| *SLC30A10* | Cohort 1 | sEOPD | Deleterious | 4 | 0.809766713 |  | 4 | 0.809766713 |
| *SLC30A10* | Cohort 2 | sLOPD | Missense | 16 | 0.668023189 |  | 18 | 0.50928517 |
| *SLC30A10* | Cohort 2 | sLOPD | Dmis | 3 | 0.159219893 |  | 3 | 0.159219893 |
| *SLC30A10* | Cohort 2 | sLOPD | Deleterious | 3 | 0.159219893 |  | 3 | 0.159219893 |
| *SLC39A14* | Cohort 1 | sEOPD | Missense | 21 | 0.611057612 |  | 21 | 0.611057612 |
| *SLC39A14* | Cohort 1 | sEOPD | Dmis | 3 | 0.779055592 |  | 3 | 0.779055592 |
| *SLC39A14* | Cohort 1 | sEOPD | Deleterious | 3 | 0.779055592 |  | 3 | 0.779055592 |
| *SLC39A14* | Cohort 2 | sLOPD | LoF | 1 | 0.178698304 |  | 1 | 0.178698304 |
| *SLC39A14* | Cohort 2 | sLOPD | Missense | 12 | 0.845426 |  | 12 | 0.845426 |
| *SLC39A14* | Cohort 2 | sLOPD | Dmis | 2 | 0.448319384 |  | 2 | 0.448319384 |
| *SLC39A14* | Cohort 2 | sLOPD | Deleterious | 3 | 0.157366442 |  | 3 | 0.157366442 |
| *SLC6A3* | Cohort 1 | sEOPD | LoF | 1 | 0.61942958 |  | 1 | 0.61942958 |
| *SLC6A3* | Cohort 1 | sEOPD | Missense | 21 | 0.609681896 |  | 23 | 0.533898408 |
| *SLC6A3* | Cohort 1 | sEOPD | Dmis | 9 | 0.81847418 |  | 9 | 0.81847418 |
| *SLC6A3* | Cohort 1 | sEOPD | Deleterious | 10 | 0.780485013 |  | 10 | 0.780485013 |
| *SLC6A3* | Cohort 2 | sLOPD | Missense | 13 | 0.610486265 |  | 15 | 0.780886991 |
| *SLC6A3* | Cohort 2 | sLOPD | Dmis | 4 | 0.311851873 |  | 4 | 0.311851873 |
| *SLC6A3* | Cohort 2 | sLOPD | Deleterious | 4 | 0.311851873 |  | 4 | 0.311851873 |
| *SPR* | Cohort 1 | sEOPD | LoF | 1 | 0.204625041 |  | 1 | 0.204625041 |
| *SPR* | Cohort 1 | sEOPD | Missense | 5 | 0.56803123 |  | 5 | 0.56803123 |
| *SPR* | Cohort 1 | sEOPD | Dmis | 1 | 0.61942958 |  | 1 | 0.61942958 |
| *SPR* | Cohort 1 | sEOPD | Deleterious | 2 | 0.273957757 |  | 2 | 0.273957757 |
| *SPR* | Cohort 2 | sLOPD | Missense | 4 | 0.524865625 |  | 4 | 0.524865625 |
| *SPR* | Cohort 2 | sLOPD | Dmis | 1 | 0.247244163 |  | 1 | 0.247244163 |
| *SPR* | Cohort 2 | sLOPD | Deleterious | 1 | 0.247244163 |  | 1 | 0.247244163 |
| *SQSTM1* | Cohort 1 | sEOPD | LoF | 3 | 0.74580283 |  | 3 | 0.74580283 |
| *SQSTM1* | Cohort 1 | sEOPD | Missense | 29 | 0.002194856 |  | 33 | 0.005410221 |
| *SQSTM1* | Cohort 1 | sEOPD | Dmis | 12 | 0.021206264 |  | 13 | 0.193906933 |
| *SQSTM1* | Cohort 1 | sEOPD | Deleterious | 15 | 0.16676745 |  | 16 | 0.439312266 |
| *SQSTM1* | Cohort 2 | sLOPD | Missense | 22 | 0.836056557 |  | 25 | 0.622035348 |
| *SQSTM1* | Cohort 2 | sLOPD | Dmis | 9 | 0.698352785 |  | 10 | 0.537366079 |
| *SQSTM1* | Cohort 2 | sLOPD | Deleterious | 9 | 0.698352785 |  | 10 | 0.537366079 |
| *TH* | Cohort 1 | sEOPD | LoF | 5 | 0.031485705 |  | 5 | 0.031485705 |
| *TH* | Cohort 1 | sEOPD | Missense | 35 | 0.519052932 |  | 40 | 0.912297707 |
| *TH* | Cohort 1 | sEOPD | Dmis | 20 | 0.594802899 |  | 22 | 0.750262476 |
| *TH* | Cohort 1 | sEOPD | Deleterious | 25 | 0.500998869 |  | 27 | 0.524281955 |
| *TH* | Cohort 2 | sLOPD | LoF | 2 | 0.394415182 |  | 2 | 0.394415182 |
| *TH* | Cohort 2 | sLOPD | Missense | 26 | 0.653398572 |  | 32 | 0.798907131 |
| *TH* | Cohort 2 | sLOPD | Dmis | 13 | 0.665591887 |  | 16 | 0.774450626 |
| *TH* | Cohort 2 | sLOPD | Deleterious | 15 | 0.712035872 |  | 18 | 0.800431017 |
| *THAP1* | Cohort 1 | sEOPD | Missense | 5 | 0.032231531 |  | 5 | 0.032231531 |
| *THAP1* | Cohort 1 | sEOPD | Dmis | 2 | 0.146077259 |  | 2 | 0.146077259 |
| *THAP1* | Cohort 1 | sEOPD | Deleterious | 2 | 0.146077259 |  | 2 | 0.146077259 |
| *THAP1* | Cohort 2 | sLOPD | Missense | 3 | 0.150354218 |  | 3 | 0.150354218 |
| *THAP1* | Cohort 2 | sLOPD | Dmis | 1 | 0.367564232 |  | 1 | 0.367564232 |
| *THAP1* | Cohort 2 | sLOPD | Deleterious | 1 | 0.367564232 |  | 1 | 0.367564232 |
| *TOR1A* | Cohort 1 | sEOPD | Missense | 14 | 0.453196377 |  | 15 | 0.475207925 |
| *TOR1A* | Cohort 1 | sEOPD | Dmis | 3 | 0.104626721 |  | 3 | 0.104626721 |
| *TOR1A* | Cohort 1 | sEOPD | Deleterious | 3 | 0.104626721 |  | 3 | 0.104626721 |
| *TOR1A* | Cohort 2 | sLOPD | Missense | 6 | 0.180187251 |  | 7 | 0.137274312 |
| *TSPOAP1* | Cohort 1 | sEOPD | Missense | 73 | 0.096447535 |  | 83 | 0.664612121 |
| *TSPOAP1* | Cohort 1 | sEOPD | Dmis | 5 | 0.379657222 |  | 6 | 0.602367487 |
| *TSPOAP1* | Cohort 1 | sEOPD | Deleterious | 5 | 0.379657222 |  | 6 | 0.602367487 |
| *TSPOAP1* | Cohort 2 | sLOPD | Missense | 76 | 0.140157314 |  | 88 | 0.460885205 |
| *TSPOAP1* | Cohort 2 | sLOPD | Dmis | 4 | 0.723219653 |  | 5 | 0.482399778 |
| *TSPOAP1* | Cohort 2 | sLOPD | Deleterious | 4 | 0.723219653 |  | 5 | 0.482399778 |
| *TUBB4A* | Cohort 1 | sEOPD | LoF | 2 | 0.524195525 |  | 2 | 0.524195525 |
| *TUBB4A* | Cohort 1 | sEOPD | Missense | 7 | 0.711664568 |  | 7 | 0.711664568 |
| *TUBB4A* | Cohort 1 | sEOPD | Dmis | 2 | 0.472869111 |  | 2 | 0.472869111 |
| *TUBB4A* | Cohort 1 | sEOPD | Deleterious | 4 | 0.592503904 |  | 4 | 0.592503904 |
| *TUBB4A* | Cohort 2 | sLOPD | Missense | 5 | 0.636649074 |  | 5 | 0.636649074 |
| *TUBB4A* | Cohort 2 | sLOPD | Dmis | 1 | 0.346181032 |  | 1 | 0.346181032 |
| *TUBB4A* | Cohort 2 | sLOPD | Deleterious | 1 | 0.346181032 |  | 1 | 0.346181032 |
| *VPS16* | Cohort 1 | sEOPD | LoF | 2 | 0.258014805 |  | 2 | 0.258014805 |
| *VPS16* | Cohort 1 | sEOPD | Missense | 44 | 0.317984587 |  | 53 | 0.221796117 |
| *VPS16* | Cohort 1 | sEOPD | Dmis | 13 | 0.724118829 |  | 16 | 0.682721958 |
| *VPS16* | Cohort 1 | sEOPD | Deleterious | 15 | 0.72068767 |  | 18 | 0.682215134 |
| *VPS16* | Cohort 2 | sLOPD | Missense | 26 | 0.049036061 |  | 33 | 0.675357509 |
| *VPS16* | Cohort 2 | sLOPD | Dmis | 9 | 0.076131902 |  | 12 | 0.518574936 |
| *VPS16* | Cohort 2 | sLOPD | Deleterious | 9 | 0.076131902 |  | 12 | 0.518574936 |
| *VPS41* | Cohort 1 | sEOPD | LoF | 4 | 0.241213482 |  | 4 | 0.241213482 |
| *VPS41* | Cohort 1 | sEOPD | Missense | 31 | 0.923643119 |  | 35 | 0.784556805 |
| *VPS41* | Cohort 1 | sEOPD | Dmis | 12 | 1 |  | 12 | 1 |
| *VPS41* | Cohort 1 | sEOPD | Deleterious | 16 | 0.874603839 |  | 16 | 0.874603839 |
| *VPS41* | Cohort 2 | sLOPD | LoF | 3 | 0.479265545 |  | 3 | 0.479265545 |
| *VPS41* | Cohort 2 | sLOPD | Missense | 18 | 0.133287491 |  | 21 | 0.132293673 |
| *VPS41* | Cohort 2 | sLOPD | Dmis | 8 | 0.320111908 |  | 8 | 0.320111908 |
| *VPS41* | Cohort 2 | sLOPD | Deleterious | 11 | 0.246790453 |  | 11 | 0.246790453 |
| *YY1* | Cohort 1 | sEOPD | Missense | 8 | 0.24076888 |  | 9 | 0.557352156 |
| *YY1* | Cohort 2 | sLOPD | Missense | 2 | 0.253743343 |  | 2 | 0.253743343 |

^a^ LoF: loss-of-function variants; Dmis: damaging missense variants (Missense variants with ReVe ≥ 0.7); Deleterious: the combination of damaging missense and loss of function variants were defined as “deleterious”. MAF: Minor allele frequency in East Asian population of gnomAD exome database and genome database; sEOPD: Sporadic early-onset Parkinson’s disease; sLOPD: Sporadic late-onset Parkinson’s disease
